# ﻿*Colletotrichum* species (Glomerellales, Glomerellaceae) causing walnut anthracnose in China

**DOI:** 10.3897/mycokeys.108.127734

**Published:** 2024-08-30

**Authors:** Lin Zhang, Lili Zhao, Chen Liang, Luhan Yu, Ying Zhang

**Affiliations:** 1 School of Ecology and Nature Conservation, Beijing Forestry University, Beijing 100083, China Beijing Forestry University Beijing China; 2 Key Laboratory of Integrated Crop Pest Management of Shandong Province, College of Plant Health and Medicine, Qingdao Agricultural University, Qingdao, Shandong 266109, China Qingdao Agricultural University Shandong China; 3 Department of Environmental Sciences, University of British Columbia, Vancouver, Canada University of British Columbia Vancouver Canada

**Keywords:** *Colletotrichum*, distribution, multi-gene phylogeny, pathogenicity, walnut anthracnose

## Abstract

*Colletotrichum* species can function as plant pathogens, saprobes or endophytes on a wide variety of plant hosts and are considered amongst the ten most significant genera of plant pathogens globally. China contributes almost half the walnut production in the world. However, *Colletotrichum* species occurring on walnut remain largely unresolved in China. To explore the *Colletotrichum* species found on walnut in China, 470 walnut fruit or leaf samples with anthracnose were collected from 14 main walnut-producing regions across seven provinces. A total of 165 *Colletotrichum* strains were isolated from these samples. The *Colletotrichum* isolates were identified, based on morphological characteristics and sequence analyses of *ACT*, *CHS-1*, *GAPDH*, ITS and *TUB2*. Twelve species, including 11 known *Colletotrichum* species (*C.boninense*, *C.citrulli*, *C.fioriniae*, *C.fructicola*, *C.godetiae*, *C.juglandicola*, *C.karsti*, *C.mengyinense*, *C.pandanicola*, *C.peakense* and *C.siamense*) and a novel species (*C.chinensis***sp. nov.**) were identified. The species distribution revealed regional prevalence as follows: *C.mengyinense* was the most dominant species in Gansu, *C.mengyinense* and *C.siamense* in Shandong, *C.chinensis* in Beijing, *C.pandanicola* in Shaanxi and *C.godetiae* in Yunnan. *Colletotrichumsiamense* was the sole species isolated in Sichuan and Xinjiang Provinces. Koch’s postulates were fulfilled, demonstrating that all 12 species cause anthracnose on walnut. This is the first report of *C.boninense*, *C.citrulli* and *C.karsti* as pathogens of walnut anthracnose worldwide.

## ﻿Introduction

Walnut (*Juglansregia* L., Juglandaceae) is an essential woody nut and oil crop cultivated worldwide, ranking first amongst the four nut types globally (Da Lio et al. 2018). Walnuts are popular fruits in China with a cultivation history of more than two thousand years (Guo 2016). In 2017, China contributed 47% of the global walnut production, maintaining the top global ranking since then (Liu et al. 2021). Due to their remarkable adaptability, extensive walnut-producing areas have been established across China (Pei and Lu 2011).

The genus *Colletotrichum* was introduced, based on the conidiomata with setae, with *C.lineola* Corda designated as the generic type (Corda 1831). The sexual morph of *Colletotrichum* was previously assigned to the genera *Gnomoniopsis* and *Glomerella* (Marin-Felix et al. 2017). With the implementation of “one fungus one name” nomenclature, *Colletotrichum* has been principally chosen to represent this genus (Réblová et al. 2016). More than 1,000 epithets have been accommodated within *Colletotrichum* (http://www.indexfungorum.org, accessed March 2024). Taxonomy of *Colletotrichum* species was confusing due to their morphological similarities and the absence of comprehensive studies using a polyphasic approach to differentiate between different species (Talhinhas and Baroncelli 2021). Morphological characteristics, however, had been considered useful in distinguishing species complexes (Cannon et al. 2012). For instance, the conidia of *C.acutatum* species complex tends to have acute ends or at least one acute end (Damm et al. 2012a), while the typical conidia of *C.boninense* species complex is cylindrical with a prominent basal scar (Damm et al. 2012b). Additionally, the conidia of *C.gloeosporioides* species complex tends to be cylindrical with round ends (Weir et al. 2012). A combination of morphological characteristics and multi-gene sequence analyses have been widely applied to define the taxonomic status in *Colletotrichum* species, which have been assigned into sixteen species complexes and some singleton species (Marin-Felix et al. 2017; Talhinhas and Baroncelli 2021; Liu et al. 2022).

*Colletotrichum* spp. comprised important plant pathogens, while some are endophytes or saprobes and could attack > 3,200 species of monocot and dicot plants (Cannon et al. 2012). Seventeen *Colletotrichum* species have been reported causing walnut anthracnose in the world, viz. *C.acutatum*, *C.aenigma*, *C.fioriniae*, *C.fructicola*, *C.gloeosporioides*, *C.godetiae*, *C.juglandicola*, *C.juglandis*, *C.kahawae*, *C.liaoningense*, *C.mengyinense*, *C.nymphaeae*, *C.pandanicola*, *C.peakense*, *C.siamense*, *C.sojae* and *C.viniferum* (He et al. 2019; Varjas et al. 2019; Mu et al. 2021; Wei et al. 2022; Cho et al. 2023; Li et al. 2023; Wang et al. 2023; Zhang et al. 2023). Several species of *Colletotrichum* have caused significant reductions in walnut production worldwide (Zhu et al. 2014; Wang et al. 2016; Da Lio et al. 2018). For instance, the causal agent of walnut anthracnose identified as belonging to the *Colletotrichum* genus led to 50–70% losses, with some walnut orchards experiencing 100% losses in nut production in France (Giraud and Verhaeghe 2015). *Colletotrichumnymphaeae* caused anthracnose on walnut in Brazil, destroyed approximately 50% of the fruits and the incidence was higher in rainy and hot summers (Savian et al. 2019).

In China, severe walnut anthracnose occurred in the orchards of Shandong Province, with the causal agents *C.gloeosporioides*, *C.siamense*, *C.fructicola* and *C.viniferum* (Zhu et al. 2014; Wang et al. 2017, 2018; He et al. 2019). The walnut leaf anthracnose caused by *C.fioriniae* led to severe losses in nut production in Hechi, Guangxi Province (Zhu et al. 2015). In addition, *Colletotrichumaenigma* caused severe fruit anthracnose in Hebei Province (Wang et al. 2021). *Colletotrichumnymphaeae* caused walnut branches anthracnose in Gansu Province (Ma et al. 2022). *Colletotrichumgloeosporioides*, *C.kahawae*, *C.nymphaeae*, *C.godetiae*, *C.fioriniae* and *C.juglandis* caused leaf spots of walnut in Hubei Province (Wei et al. 2022). Additionally, *Colletotrichumgodetiae* caused severe anthracnose of walnut in Shaanxi and Yunnan Provinces with diseased fruits over 60% in the orchards (Wang et al. 2023).

Despite these studies on walnut anthracnose caused by *Colletotrichum* spp. from different regions in China, a comprehensive investigation into the species composition, geographic distribution and pathogenicity of these species is lacking. The aims of this study were to: (i) determine the species composition and geographic distribution of *Colletotrichum* spp. associated with walnut anthracnose in the principal production regions of China; and (ii) evaluate the pathogenicity of the *Colletotrichum* spp. by Koch’s postulates.

## ﻿Materials and methods

### ﻿Sample collection and fungal isolation

During 2021–2023, a total of 470 fruit or leaf samples with anthracnose were collected from 14 primary walnut-producing areas in seven provinces (including Shandong, Yunnan, Sichuan, Shaanxi, Gansu, Xinjiang and Beijing) in China. Amongst these samples, there were 342 fruit samples and 128 leaf samples. Fragments (0.5 cm × 0.5 cm) of walnut, including the leaves and fruits, were cut aseptically from the margin of the disease lesion. The fragments were surface sterilised with 75% ethanol for 30 s, rinsed three times with sterile distilled water and dried on sterilised filter paper. Finally, the fragments were incubated on malt extract agar (MEA) for isolation of fungal strains (Fu et al. 2019). Petri dishes containing MEA with the fungal strains were incubated in the dark at 25 °C until the fungal colonies were observed. Hyphal tips resembling *Colletotrichum* colonies were transferred to Petri dishes with MEA.

### ﻿DNA extraction, PCR amplification and sequencing

DNA was extracted from mycelia grown on MEA plates with a CTAB plant genome DNA fast extraction kit (Aidlab Biotechnologies Co., Ltd, Beijing, China) and stored at -20 °C until further use. Five loci including the 5.8S nuclear ribosomal gene with the two flanking internal transcribed spacers (ITS), a 200-bp intron of the glyceraldehyde-3-phosphate dehydrogenase (GAPDH) and partial actin (ACT), beta-tubulin (TUB2) and chitin synthase (*CHS-1*) were amplified using the primer pairs ITS1/ITS4 (White et al. 1990; Gardes and Bruns 1993), GDF1/GDR1 (Guerber et al. 2003), ACT-512F/ACT-783R (Carbone and Kohn 1999), T1/Bt2b (Glass and Donaldson 1995; O’Donnell and Cigelnik 1997) and CHS-79F/CHS-345R (Carbone and Kohn 1999), respectively.

PCR amplification and sequencing were conducted following the protocols established by Fu et al. (2019). PCR amplicons were purified and sequenced at BGI Tech Solutions (Beijing Liuhe) Co., Limited (Beijing, China). Forward and reverse were assembled to obtain a consensus sequence using DNAMAN (v. 6.0.3.99; Lynnon Biosoft). Sequences generated in this study were deposited in GenBank (Suppl. material 1).

### ﻿Phylogenetic analyses

DNA sequences of concatenated *ACT*, *CHS-1*, *GAPDH*, ITS and *TUB2* loci were analysed to investigate the phylogenetic relationships amongst *Colletotrichum* species with DNA sequences available from GenBank (http://www.ncbi.nlm.nih.gov/genbank/accessed March 2024) (Suppl. material 1). Multiple sequences were aligned using the MAFFT v.7.110 (http://mafft.cbrc.jp/alignment/server/ accessed March 2024) and adjusted manually in MEGA v.7.0 (Kumar et al. 2016). Gaps were manually adjusted to optimise the alignment (Tamura et al. 2013).

Phylogenetic analyses of Maximum Likelihood (ML), Bayesian Inference (BI) and Maximum parsimony (MP) were performed. Maximum Likelihood analyses were constructed on the RAxML-HPC BlackBox 8.2.10 (Stamatakis 2014) using the GTR+GAMMA model with 1,000 bootstrap replicates. The Bayesian phylogenetic analysis was performed using a Markov Chain Monte Carlo (MCMC) algorithm in MrBayes v. 3.2.6 (Ronquist et al. 2012). Four MCMC chains were run from random trees and trees were sampled by each 1,000^th^ generation. The first 25% of the trees of MCMC sampling were discarded as burn-in and posterior probabilities (PP) were determined from the remaining trees. Maximum parsimony (MP) analysis, based on the concatenated dataset, was conducted in PAUP* v. 4.0b10 with the default options (Swofford 2002). Ambiguous regions in the alignment were excluded and gaps were treated as missing data. Clade stability was evaluated in a bootstrap analysis with 1,000 replicates with maxtrees set to 1,000 with other default parameters used as implemented in PAUP* (Hillis and Bull 1993). Other measures used to evaluate parsimony scores included the consistency index (CI), rescaled consistency (RC), homoplasy index (HI) and retention index (RI). The phylogenetic trees were configured in FigTree v. 1.4.4 (http://tree.bio.ed.ac.uk/software/figtree) and edited using Adobe Illustrator CC2020 (Adobe Systems Inc., USA).

### ﻿Morphological characterisation

To assess the colony characteristics, mycelial plugs (8 mm in diameter) were transferred from the growing edges of 7-day-old colonies on to PDA and MEA and incubated at 25 °C in darkness. Colony diameters were measured after 7 days of incubation and were used to calculate the mycelium growth rate (Zhang et al. 2023). Morphology and colony characteristics were determined following the methods described by Damm et al. (2012a). Appressoria were induced on slide cultures according to the protocol established by Weir et al. (2012). The shape, colour and size of conidia, conidiophores, setae, conidiogenous cells and appressoria were measured by at least 20 measurements using a microscope (Nikon Eclipse E600) (Zhang et al. 2023). Fungal isolates and specimens were deposited at Beijing Forestry University, with duplicates at the China General Microbiological Culture Collection Center (CGMCC; https://www.cgmcc.net/english, accessed March 2024).

### ﻿Prevalence

To determine the abundance of *Colletotrichum* species in sampled provinces, the isolation rate (R^I^) for each species was calculated using the formula: R^I^ % = (N^s^/N^t^) × 100, where N^s^ represents the number of isolates from the same species and N^t^ is the total number of isolates from each sample-collected province (Fu et al. 2019).

### ﻿Pathogenicity test and virulence on walnut tissues

The pathogenicity of all isolated species was examined on walnut fruits and leaves. Mycelial plugs derived from representative isolates obtained in this study were utilised for pathogenicity test. Isolates of all species were incubated on MEA plates for 7 days prior to inoculation.

The pathogenicity test was performed on detached living walnut fruits and leaves. Briefly, fruits and leaves were washed with sterilised water and surfaces sterilised with 75% ethanol for 1 min. The fruits and leaves were inoculated using the spore suspension and non-wound inoculation methods (Fu et al. 2019; Zhang et al. 2023). For inoculation, an aliquot of 20 μl of spore suspension (1.0 × 10^6^ conidia per ml) was inoculated on to fruits and leaves without wounding them. Eight replicates were used for each treatment. Fruits and leaves, inoculated by sterilised water, served as the negative control. The inoculated detached fruits and leaves were incubated under 25 °C with 12/12 h light/dark photoperiod. Pathogenicity was determined by measuring the lesion length of fruits and leaves after 10 days’ incubation. To fulfil Koch’s postulates, the fungal pathogens were re-isolated from the lesion and identified, based on morphology and DNA sequences.

Mean comparisons were conducted using Tukey’s honest significant difference (HSD) test (α = 0.05) in R (Version 3.2.2, R Inc. Auckland, NZL).

## ﻿Results

### ﻿*Colletotrichum* species associated with walnut anthracnose

**Figure 1.**
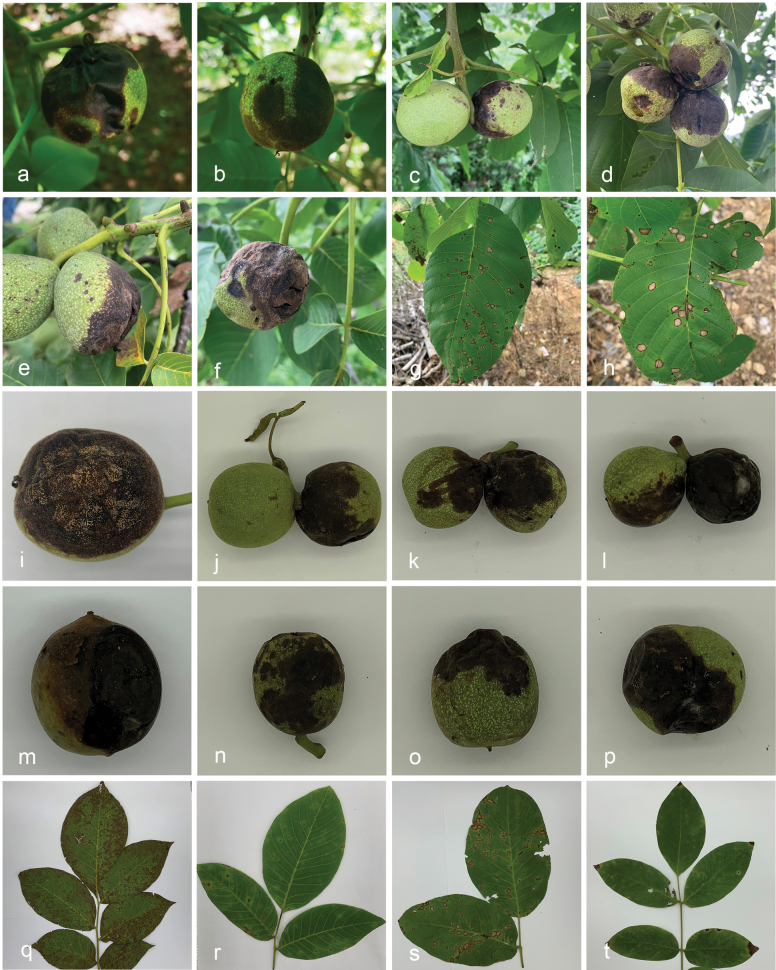
Representative symptoms of walnut anthracnose on leaves and fruits **a–f, i–p** symptoms on fruits of *Juglansregia***g, h, q–t** symptoms on leaves of *Juglansregia*.

**Figure 2.**
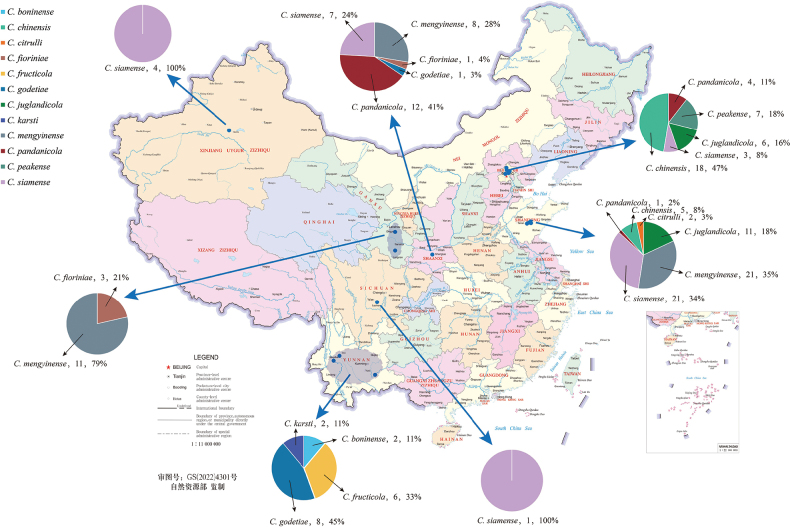
Map of China indicating locations where walnut trees were sampled and the species of *Colletotrichum* obtained from each province. The 12 species of *Colletotrichum* are indicated.

During 2021–2023, a total of 342 fruit samples and 128 leaf samples from diseased walnut trees exhibiting anthracnose were collected in seven provinces (Beijing, Shandong, Yunnan, Sichuan, Shaanxi, Gansu and Xinjiang) of China. (Figs 1, 2). A total of 165 *Colletotrichum* strains were isolated from these samples. The occurrence of 12 *Colletotrichum* species is summarised in Suppl. material 2.

### ﻿Multi-locus phylogenetic analyses

**Figure 3.**
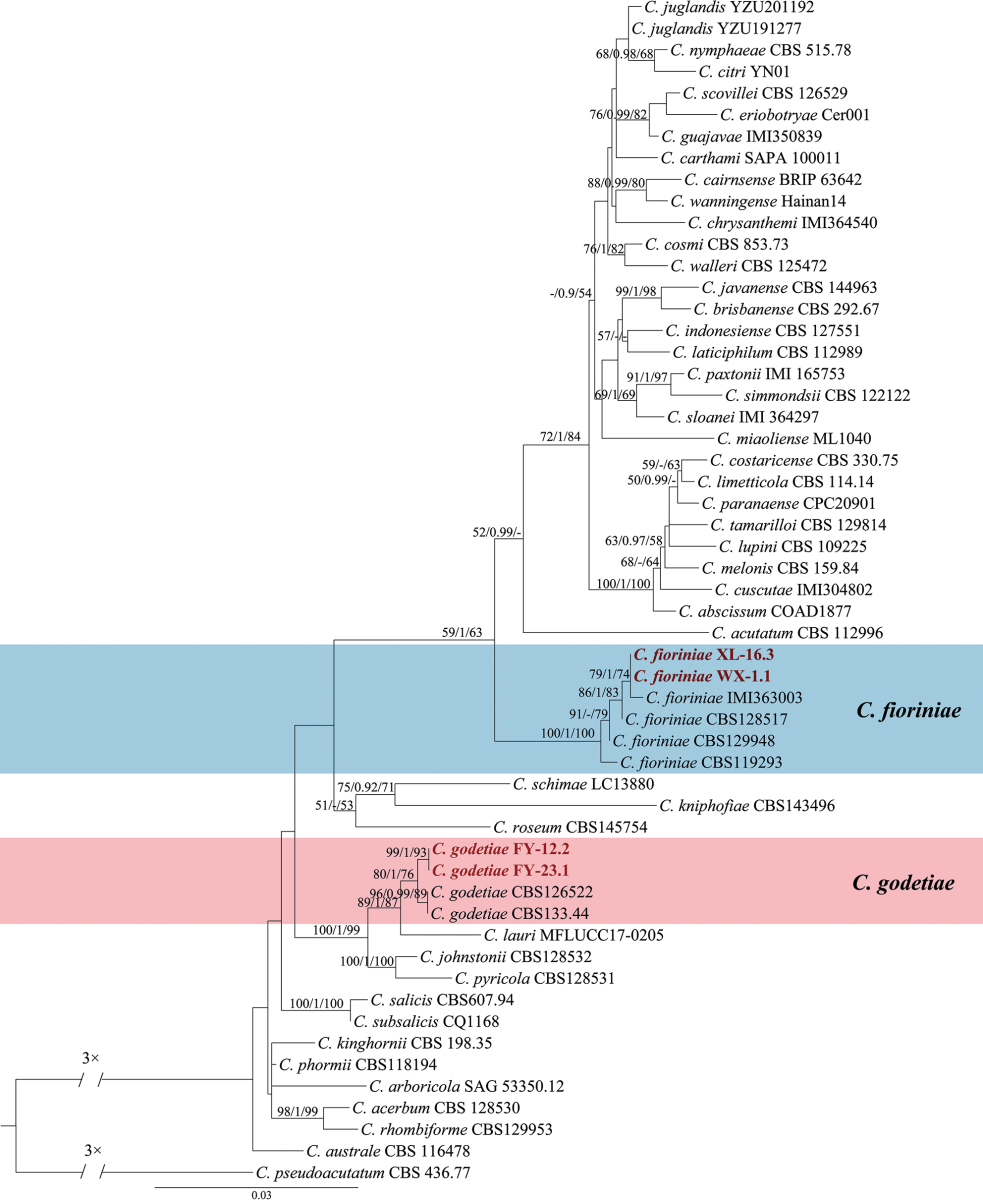
Maximum Likelihood tree generated from sequence analysis of the concatenated *ACT*, *CHS-1*, *GAPDH*, ITS and *TUB2* genes dataset of *C.acutatum* species complex. The species *C.pseudoacutatum* CBS 436.77 was selected as an outgroup. Bayesian posterior probability (PP ≥ 0.90), MP bootstrap support values (ML ≥ 50%) and RAxML bootstrap support values (ML ≥ 50%) were shown at the nodes (ML/PP/MP).

**Figure 4.**
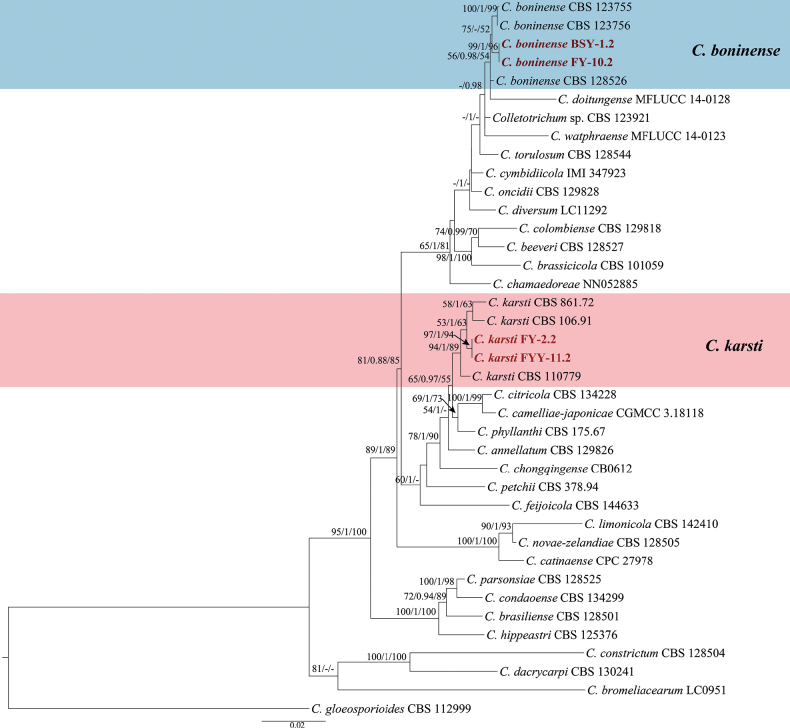
Maximum Likelihood tree generated from sequence analysis of the concatenated *ACT*, *CHS-1*, *GAPDH*, ITS and *TUB2* genes dataset of *C.boninense* species complex. The species *C.gloeosporioides* CBS 112999 was selected as an outgroup. Bayesian posterior probability (PP ≥ 0.90), MP bootstrap support values (ML ≥ 50%) and RAxML bootstrap support values (ML ≥ 50%) were shown at the nodes (ML/PP/MP).

**Figure 5.**
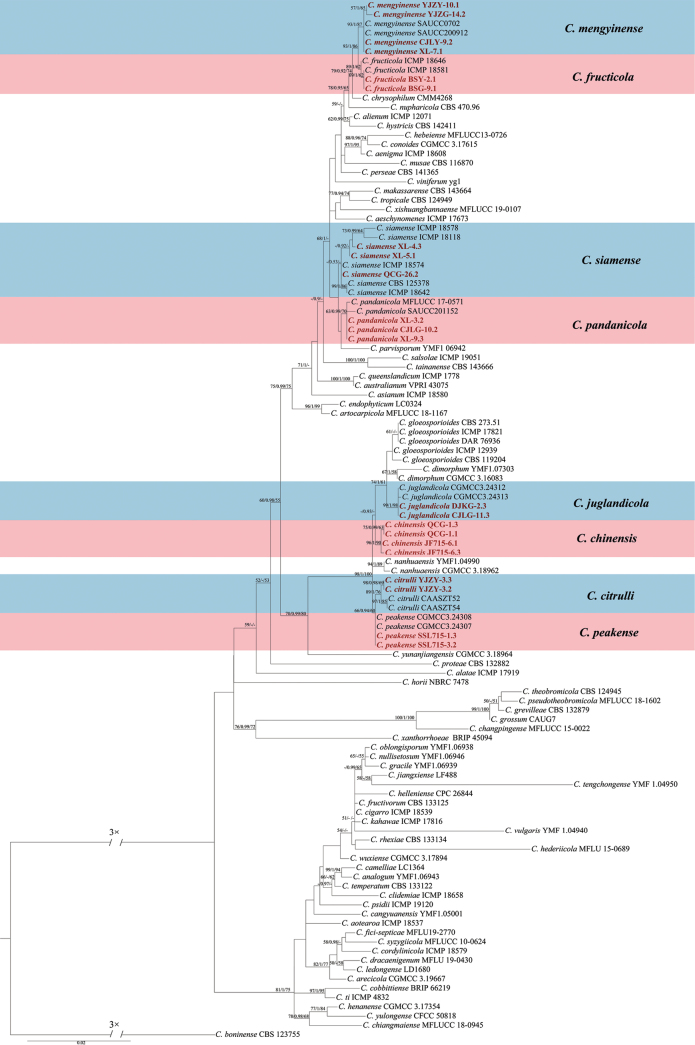
Maximum Likelihood tree generated from sequence analysis of the concatenated *ACT*, *CHS-1*, *GAPDH*, ITS and *TUB2* genes dataset of *C.gloeosporioides* species complex. The species *C.boninense* CBS 123755 was selected as an outgroup. Bayesian posterior probability (PP ≥ 0.90), MP bootstrap support values (ML ≥ 50%) and RAxML bootstrap support values (ML ≥ 50%) were shown at the nodes (ML/PP/MP).

Based on the results of BLAST in GenBank, 31 representative isolates together with 149 previously described species (Suppl. material 2) were subjected to multi-locus phylogenetic analyses with concatenated *ACT*, *CHS-1*, *GAPDH*, ITS and *TUB2* sequences for those belonging to the *C.gloeosporioides*, *C.boninense* and *C.acutatum* species complex. The results indicated that these 31 isolates clustered together with 12 species (Figs 3–5).

The concatenated *ACT*, *CHS-1*, *GAPDH*, ITS and *TUB2* dataset (1,772 characters with 251 parsimony-informative characters) from 55 ingroup isolates of *Colletotrichumacutatum* species complex was used for phylogenetic analysis. The heuristic search with random addition of taxa (1,000 replicates) generated 5,000 most parsimonious trees (Length = 962, CI = 0.671, HI = 0.329, RI = 0.830, RC = 0.557). The topologies obtained from the Maximum Parsimony, Maximum Likelihood and Bayesian analysis were comparable. In three analyses (ML, Bayesian and MP), four isolates clustered in two clades corresponding to *C.fioriniae* (2 isolates) and *C.godetiae* (2 isolates) (Fig. 3).

The concatenated *ACT*, *CHS-1*, *GAPDH*, ITS and *TUB2* dataset (1,875 characters with 339 parsimony-informative characters) from 39 ingroup isolates of *Colletotrichumboninense* species complex was used for phylogenetic analysis. The heuristic search with random addition of taxa (1,000 replicates) generated 5,000 most parsimonious trees (Length = 1283, CI = 0.672, HI = 0.327, RI = 0.728, RC = 0.490). The topologies obtained from the Maximum Parsimony, Maximum Likelihood and Bayesian analysis were comparable. In three analyses (ML, Bayesian and MP), four isolates clustered in two clades corresponding to *C.boninense* (2 isolates) and *C.karsti* (2 isolates) (Fig. 4).

The concatenated *ACT*, *CHS-1*, *GAPDH*, ITS and *TUB2* dataset (1,959 characters with 372 parsimony-informative characters) from 106 ingroup isolates of *Colletotrichumgloeosporioides* species complex was used for phylogenetic analysis. The heuristic search with random addition of taxa (1,000 replicates) generated 5,000 most parsimonious trees (Length = 1510, CI = 0.580, HI = 0.420, RI = 0.811, RC = 0.470). The topologies obtained from the Maximum Parsimony, Maximum Likelihood and Bayesian analysis were comparable. In the phylogenetic tree constructed for the isolates in the *C.gloeosporioides* complex, 23 isolates clustered in eight clades corresponding to *C.citrulli* (2 isolates), *C.fructicola* (2 isolates), *C.juglandicola* (2 isolates), *C.mengyinense* (4 isolates), *C.pandanicola* (4 isolates), *C.peakense* (2 isolates) and *C.siamense* (3 isolates). Noticeably, four isolates (CGMCC 3.25209, CGMCC 3.25210, CGMCC 3.25211 and CGMCC 3.25212) clustered distantly from any known species in the complex and are herein described as a new taxon, namely *C.chinensis*, based on the guidelines established in Chethana et al. (2021) and Jayawardena et al. (2021) (Fig. 5).

### ﻿Morphological investigation

Colonies of the representative isolates were selected to observe their morphological characteristics (Suppl. materials 3, 4). Some differences in colony morphology were obviously observed amongst the 12 species. Abundant orange conidial masses were often observed in the centre of the colonies. A significant difference in growth rate across 12 *Colletotrichum* species was observed (Suppl. material 4). Colonies diam. on MEA, the mycelial growth rate of *C.mengyinense* was the highest, with an average of 80.3 ± 0.6 mm after 7 days. This was followed by *C.citrulli* (78.8 ± 6.3 mm), *C.pandanicola* (76.3 ± 1.5 mm), *C.peakense* (76.3 ± 2.6 mm) and *C.chinensis* (75.7 ± 0.6 mm). The growth rate of *C.godetiae* was the slowest (48.7 ± 2.1 mm) (Suppl. material 4). Colonies diam. on PDA, the mycelial growth rate of *C.mengyinense* was the highest, with an average of 82.3 ± 1.5 mm after 7 days, followed by *C.siamense* (82.0 ± 0 mm) and *C.chinensis* (80.0 ± 0 mm). The growth rate of *C.juglandicola* was the slowest (49.1 ± 1.9 mm) (Suppl. material 4).

#### ﻿ Colletotrichum chinensis

Taxon classificationFungiGlomerellalesGlomerellaceae

Y. Zhang ter & L. Zhang sp. nov.

6E6EEE6E-5879-5D8A-B509-7AC27F1A67D5

Index Fungorum: IF901166

Facesoffungi Number: FoF14886

Fig. 6

##### Holotype.

QCG-1.

##### Etymology.

Named after China where the fungus was collected.

##### Description.

Associated with walnut fruit and leaf anthracnose. Sexual morph not observed. Asexual morph developed on MEA. ***Conidiomata*** acervular, conidiophores hyaline, smooth-walled, septate, branched. ***Setae*** medium to dark brown, smooth to finely verruculose close to the tip, the tip rounded, 1–3 aseptate, 39.2–118.7 μm long. ***Conidiogenous cells*** subcylindrical, straight to curved, 16.7–30.0 × 2.3–3.7 μm (mean ± SD = 22.2 ± 0.6 × 3.2 ± 0.1 μm, n = 30). ***Conidia*** hyaline, smooth-walled, subcylindrical, both ends round, 1–3-guttulate, contents granular, 13.7–18.5 × 4.4–5.9 μm (mean ± SD = 16.4 ± 1.0 × 5.0 ± 0.3 μm, L/W radio = 3.3, n = 100).

**Figure 6.**
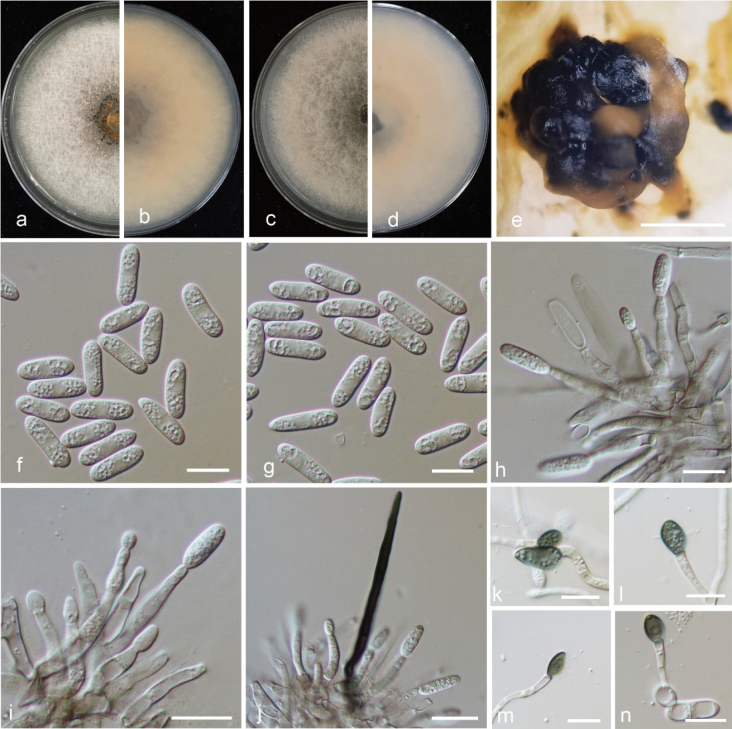
Morphological characteristics of *Colletotrichumchinensis***a, b** front and back view, respectively, of 7-d-old MEA culture **c, d** front and back view, respectively, of 7-d-old PDA culture **e** conidiomata **f, g** conidia **h, i** conidiophores **j** setae **k–n** appressoria **a–n** isolate QCG-1.1. Scale bars: 10 μm (**f–n**); 500 μm (**e**).

##### Culture characteristics.

Colonies on MEA flat with entire margin, surface pale pink, covered with felty white aerial mycelium aerial; reverse rosy buff to honey-coloured, growth rate 75–76 mm diam. in 7 d. Colonies on PDA flat with entire margin, surface pale pink, covered with felty white or grey aerial mycelium, grey aerial mycelium in the centre; reverse buff, rosy buff to honey-coloured, growth rate 79–80 mm diam. in 7 d. ***Appressoria*** produced on slide culture from conidia, medium to dark brown, variable in shape, often smooth-walled, subglobose, ovate to broadly elliptical in outline, 7.3–12.0 × 4.7–6.7 μm (mean ± SD = 9.5 ± 0.2 × 5.8 ± 0.1 μm, L/W radio = 1.6, n = 40).

##### Material examined.

China, Shandong Province, Taian City, on fruit of *Juglansregia* L., 29 July 2022, Y. Zhang, L. Zhang and L.L. Zhao (holotype, QCG-1, culture ex-type, QCG-1.1 = CGMCC 3.25209; culture QCG-1.3 = CGMCC 3.25210); Beijing City, on fruit of *Juglansregia* L., 15 July 2022, Y. Zhang, L. Zhang and L.L. Zhao (JF715-6, culture, JF715-6.1 = CGMCC 3.25211; culture, JF715-6.3 = CGMCC 3.25212).

##### Notes.

Phylogenetic analysis of the concatenated set of nucleotides from five loci indicated that *Colletotrichumchinensis* nested in the clade of *C.gloeosporioides* species complex and was closely related to *C.citrulli*, *C.dimorphum*, *C.gloeosporioides*, *C.juglandicola*, *C.nanhuaensis* and *C.peakense* (Cannon et al. 2008; Guo et al. 2022; Yu et al. 2022; Zhang et al. 2023). Morphologically, the strikingly longer conidia or appressoria of *C.chinensis* could be readily distinguishable from *C.citrulli*, *C.dimorphum*, *C.gloeosporioides*, *C.juglandicola*, *C.nanhuaensis* or *C.peakense. Colletotrichumcitrulli*, *C.dimorphum* and *C.nanhuaensis* were originally reported from *AgeratinaAdenophora* (Spreng.) King & H.Rob. and *Citrulluslanatus* (Thunb.) Matsum & Nakai, respectively in China (Guo et al. 2022). *Colletotrichumjuglandicola* and *C.peakense* had been reported from *Juglansregia* L. as new species in China (Zhang et al. 2023). Thus, *Colletotrichumchinensis* was identified as a new species in this study, which caused anthracnose of *Juglansregia*.

### ﻿Prevalence

In this study, *Colletotrichummengyinense* was the dominant species (40/165, 24.2%), followed by *C.siamense* (36/165, 21.8%), *C.chinensis* (23/165, 13.9%), *C.pandanicola* (17/165, 10.3%), *C.juglandicola* (17/165, 10.3%), *C.godetiae* (9/165, 5.5%), *C.peakense* (7/165, 4.2%), *C.fructicola* (6/165, 3.6%), *C.fioriniae* (4/165, 2.4%), *C.boninense* (2/165, 1.3%), *C.karsti* (2/165, 1.2%) and *C.citrulli* (2/165, 1.2%) (Suppl. material 1), of which, *C.mengyinense* was the most dominant species in Gansu Province (79%), *C.chinensis* in Beijing (47%), *C.pandanicola* in Shaanxi (41%) and *C.godetiae* (45%) in Yunnan. *Colletotrichummengyinense* and *C.siamense* were prevalent species in Shandong Province (35%). *Colletotrichumsiamense* was the only species isolated in Sichuan and Xinjiang Provinces (Fig. 2).

### ﻿Pathogenicity test and virulence on walnut tissues

**Figure 7.**
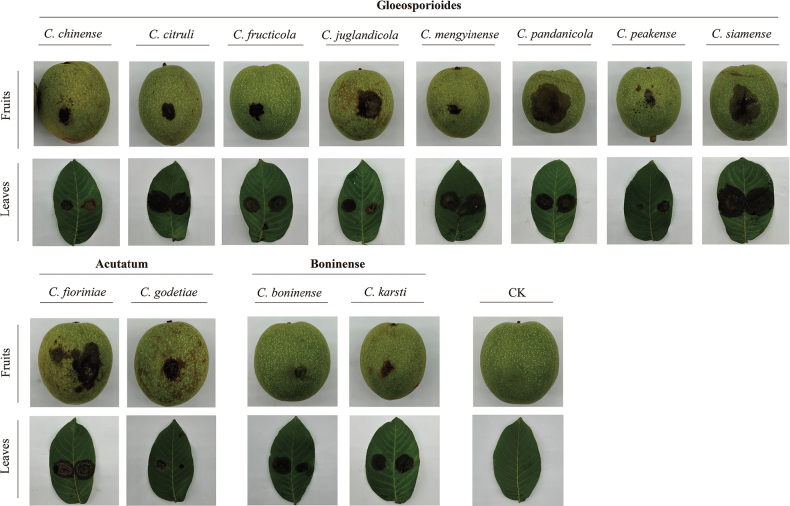
Symptoms of 12 *Colletotrichum* species inoculated on walnut fruits (*Juglansregia* L.) and leaves at 10 days.

Koch’s postulate tests on twelve *Colletotrichum* species indicated that all of them could cause walnut anthracnose. Necrotic lesions and typical orange conidial masses were observed from the inoculated site on fruits and leaves after ten days’ inoculation, whereas all control fruits remained healthy (Fig. 7). The fruit lesion length in the treatments inoculating *C.fioriniae* (mean ± SD = 19.2 ± 7.3 mm), *C.pandanicola* (mean ± SD = 17.1 ± 7.4 mm), *C.siamense* (mean ± SD = 13.8 ± 6.6 mm) and *C.juglandicola* (mean ± SD = 11.9 ± 3.0 mm) were significantly higher than those observed in all other treatments (*P* < 0.05) (Suppl. material 4). The leaf lesion length in the treatments inoculating *C.fioriniae* (mean ± SD = 23.3 ± 2.1 mm), *C.pandanicola* (mean ± SD = 22.4 ± 4.4 mm), *C.siamense* (mean ± SD = 21.8 ± 8.2 mm), *C.fructicola* (mean ± SD = 20.6 ± 1.1 mm), *C.citrulli* (mean ± SD = 20.3 ± 6.0 mm) and *C.mengyinense* (mean ± SD = 19.4 ± 3.6 mm) were significantly higher than all other treatments (*P* < 0.05) (Suppl. material 4). All these twelve inoculated *Colletotrichum* species were re-isolated from the necrotic fruits and leaves, thereby fulfilling Koch’s postulates.

## ﻿Discussion

In total, twelve *Colletotrichum* species within three *Colletotrichum* species complexes (namely *C.acutatum*, *C.boninense* and *C.gloeosporioides*) were identified. *Colletotrichumchinensis* was described as a species new to science in this study, while *C.boninense*, *C.citrulli* and *C.karsti* were reported from walnut for the first time. Both *C.boninense* and *C.karsti* belong to the *C.boninense* species complex. *Colletotrichumboninense* species complex comprised 29 species, some of which had a diverse host range (Talhinhas and Baroncelli 2021; Liu et al. 2022). For instance, *Colletotrichumkarsti* had been reported on more than 60 plant species worldwide (Talhinhas and Baroncelli 2021). However, this was the first reported of the members of *C.boninense* species complex on walnut.

All these twelve *Colletotrichum* species, isolated in this study, caused the typical symptoms of anthracnose on walnut, which led to the eventual mortality of the fruits or leaves. *Colletotrichumfioriniae*, *C.pandanicola*, *C.siamense* and *C.juglandicola* were more severe on walnut fruits than other *Colletotrichum* species, while *C.fioriniae*, *C.pandanicola*, *C.siamense*, *C.fructicola*, *C.citrulli* and *C.mengyinense* were more severe on leaves. *Colletotrichumgloeosporioides* s. s. had been reported as a more severe causal agent of walnut anthracnose than most other species in Beijing (Li et al. 2023). However, it was absent from the current study.

The geographical distribution of *Colletotrichum* spp. in China exhibited a distinct regional prevalence. For instance, *C.mengyinense* prevailed in Gansu Province, co-existing with *C.siamense* in Shandong, *C.chinensis* in Beijing, *C.pandanicola* in Shaanxi and *C.godetiae* in Yunnan. Notably, *Colletotrichumsiamense* was the only species isolated in Sichuan and Xinjiang Provinces. The causal agent of walnut anthracnose appeared to vary across different sampling sites. Comparable results were documented for anthracnose diseases in *Pyrus* spp., indicating variations in species distribution and occurrence across different regions (Fu et al. 2019). The potential explanation is that the fungal susceptibility is greatly affected by the host cultivars examined, environmental conditions, weather and farming practices (Dowling et al. 2020). For instance, disease severity diminishes when temperatures exceed 35 °C, while increased precipitation can boost disease development (Iqra et al. 2022).

A variety of management methods, including cultural, biological control and chemical control, have been tried over the years to manage *Colletotrichum* spp. infecting fruit crops (Dowling et al. 2020). Despite these efforts, chemical control remains the primary method for controlling these diseases. Future research should focus on including more isolates from walnuts across different regions of China to thoroughly investigate the distribution and diversity of *Colletotrichum* species. Additionally, it should evaluate the effectiveness of biological and chemical control agents on the growth of anthracnose pathogens in the field conditions.

## Supplementary Material

XML Treatment for Colletotrichum chinensis

## ﻿Additional information

### Author ORCIDs

Lin Zhang https://orcid.org/0009-0002-6325-1440

Lili Zhao https://orcid.org/0000-0003-1451-3301

Ying Zhang https://orcid.org/0000-0001-8817-6032

### Data availability

All of the data that support the findings of this study are available in the main text or Supplementary Information.

## Supplementary materials

Supplementary material 1 GenBank accession numbers of isolates included in this study Data type docx Explanation note Newly-generated sequences are in bold. https://binary.pensoft.net/file/1124106This dataset is made available under the Open Database License (http://opendatacommons.org/licenses/odbl/1.0/). The Open Database License (ODbL) is a license agreement intended to allow users to freely share, modify, and use this Dataset while maintaining this same freedom for others, provided that the original source and author(s) are credited.Lin Zhang, Lili Zhao, Chen Liang, Luhan Yu, Ying Zhang

Supplementary material 2 The occurrence of 12 *Colletotrichum* species Data type docx https://binary.pensoft.net/file/1124107This dataset is made available under the Open Database License (http://opendatacommons.org/licenses/odbl/1.0/). The Open Database License (ODbL) is a license agreement intended to allow users to freely share, modify, and use this Dataset while maintaining this same freedom for others, provided that the original source and author(s) are credited.Lin Zhang, Lili Zhao, Chen Liang, Luhan Yu, Ying Zhang

Supplementary material 3 Morphological comparisons of twelve *Colletotrichum* species in this study and their hosts in previously reported Data type docx https://binary.pensoft.net/file/1124108This dataset is made available under the Open Database License (http://opendatacommons.org/licenses/odbl/1.0/). The Open Database License (ODbL) is a license agreement intended to allow users to freely share, modify, and use this Dataset while maintaining this same freedom for others, provided that the original source and author(s) are credited.Lin Zhang, Lili Zhao, Chen Liang, Luhan Yu, Ying Zhang

Supplementary material 4 Mycelial diameter of twelve *Colletotrichum* species and lesion lengths formed on walnut fruits and leaves Data type docx https://binary.pensoft.net/file/1124109This dataset is made available under the Open Database License (http://opendatacommons.org/licenses/odbl/1.0/). The Open Database License (ODbL) is a license agreement intended to allow users to freely share, modify, and use this Dataset while maintaining this same freedom for others, provided that the original source and author(s) are credited.Lin Zhang, Lili Zhao, Chen Liang, Luhan Yu, Ying Zhang

## References

[B1] Cannon PF Buddie AG Bridge PD (2008) The typification of *Colletotrichumgloeosporioides.* Mycotaxon 104: 189–204.

[B2] Cannon PF Damm U Johnston PR Weir BS (2012) *Colletotrichum* - current status and future directions. Studies in Mycology 73: 181–213. 10.3114/sim0014 23136460 PMC3458418

[B3] Carbone I Kohn LM (1999) A method for designing primer sets for speciation studies in filamentous ascomycetes. Mycologia 91(3): 553–556. 10.1080/00275514.1999.12061051

[B4] Chethana KWT Manawasinghe IS Hurdeal VG Bhunjun CS Appadoo MA Gentekaki E Raspé O Promputtha I Hyde KD (2021) What are fungal species and how to delineate them? Fungal Diversity 109(1): 1–25. 10.1007/s13225-021-00483-9

[B5] Cho SE Oh JY Lee DH Kim CW (2023) First Report of Anthracnose on *Juglansregia* Caused by *Colletotrichumsiamense* in Korea. Plant Disease 107(1): 218. 10.1094/PDIS-02-22-0267-PDN

[B6] Corda ACI (1831) In: Sturm J (Ed.) Deutschlands Flora in Abbildungen nach der Natur mit Beschreibungen.Sturm, Nürnberg 3(12), 33–64.

[B7] Da Lio D Cobo-Díaz JF Masson C Chalopin M Kebe D Giraud M Verhaeghe A Nodet P Sarrocco S Le Floch G Baroncelli R (2018) Combined metabarcoding and multi-locus approach for genetic characterization of *Colletotrichum* species associated with common walnut (*Juglansregia*) anthracnose in France. Scientific Reports 8(1): 10765. 10.1038/s41598-018-29027-z 30018385 PMC6050315

[B8] Damm U Cannon PF Woudenberg JHC Crous PW (2012a) The *Colletotrichumacutatum* species complex. Studies in Mycology 73: 37–113. 10.3114/sim0010 23136458 PMC3458416

[B9] Damm U Cannon PF Woudenberg JHC Johnston PR Weir BS Tan YP Shivas RG Crous PW (2012b) The *Colletotrichumboninense* species complex. Studies in Mycology 73: 1–36. 10.3114/sim0002 23136457 PMC3458415

[B10] Dowling M Peres N Villani S Schnabel G (2020) Managing *Colletotrichum* on fruit crops: A “complex” challenge. Plant Disease 104(9): 2301–2316. 10.1094/PDIS-11-19-2378-FE 32689886

[B11] Fu M Crous PW Bai Q Zhang PF Xiang J Guo YS Zhao FF Yang MM Hong N Xu WX Wang GP (2019) *Colletotrichum* species associated with anthracnose of *Pyrus* spp. in China. Persoonia - Molecular Phylogeny and Evolution of Fungi 42: 1–35. 10.3767/persoonia.2019.42.01 PMC6712541 31551612

[B12] Gardes M Bruns TD (1993) ITS primers with enhanced specificity for basidiomycetes-application to the identification of mycorrhizae and rusts. Molecular Ecology 2(2): 113–118. 10.1111/j.1365-294X.1993.tb00005.x 8180733

[B13] Giraud M Verhaeghe A (2015) Fiche bio-agresseur: Anthracnose à *Colletotrichum* sp. en verger de noyers. Arboric. Fruitière. 2.

[B14] Glass NL Donaldson GC (1995) Development of primer sets designed for use with PCR to amplify conserved genes from filamentous ascomycetes. Applied and Environmental Microbiology 61(4): 1323–1330. 10.1128/aem.61.4.1323-1330.1995 7747954 PMC167388

[B15] Guerber JC Liu B Correll JC Johnston PR (2003) Characterization of diversity in *Colletotrichumacutatum* sensu lato by sequence analysis of two gene introns, mtDNA and intron RFLPs, and mating compatibility. Mycologia 95(5): 872–895. 10.1080/15572536.2004.11833047 21148995

[B16] Guo R (2016) Occurrence and control trend of main d es and pests of walnut in Baoji city. Northwest A&F University, China.

[B17] Guo Z Luo CX Wu HJ Peng B Kang BS Liu LM Zhang M Gu QS (2022) *Colletotrichum* species associated with anthracnose disease of watermelon (*Citrulluslanatus*) in China. Journal of Fungi (Basel, Switzerland) 8(8): 790. 10.3390/jof8080790 36012779 PMC9410023

[B18] He LF Li XX Gao YY Li BX Mu W Liu F (2019) Characterization and fungicide sensitivity of *Colletotrichum* spp. from different hosts in Shandong, China. Plant Disease 103(1): 34–43. 10.1094/PDIS-04-18-0597-RE 30388064

[B19] Hillis DM Bull JJ (1993) An empirical test of bootstrapping as a method for assessing confidence in phylogenetic analysis. Systematic Biology 42(2): 182–192. 10.1093/sysbio/42.2.182

[B20] Iqra ul Haq I Waseem Khan Qadri R Amrao L Ijaz S (2022) Effect of environmental conditions (temperature and precipitation) on severity of guava die-back caused by *Colletotrichum* spp. under climatic conditions of Pakistan. Journal of Plant Pathology 104: 1–12. 10.1007/s42161-021-00968-1

[B21] Jayawardena RS Hyde KD de Farias ARG Bhunjun CS Ferdinandez HS Manamgoda DS Udayanga D Herath IS Thambugala KM Manawasinghe IS Gajanayake AJ Samarakoon BC Bundhun D Gomdola D Huanraluek N Sun YR Tang X Promputtha I Thines M (2021) What is a species in fungal plant pathogens? Fungal Diversity 109(1): 239–266. 10.1007/s13225-021-00484-8

[B22] Kumar S Stecher G Tamura K (2016) MEGA7: Molecular evolutionary genetics analysis version 7.0 for bigger datasets. Molecular Biology and Evolution 33(7): 1870–1874. 10.1093/molbev/msw054 27004904 PMC8210823

[B23] Li FX Chen JW Chen Q Liu ZY Sun JY Yan YT Zhang HX Bi Y (2023) Identification, pathogenicity, and sensitivity to fungicide of *Colletotrichum* species that causes walnut anthracnose in Beijing. Agronomy (Basel) 13(1): 214. 10.3390/agronomy13010214

[B24] Liu MZ Li CH Cao CM Wang LQ Li XP Che J Yang HM Zhang XW Zhao HY He GZ Liu XD (2021) Walnut fruit processing equipment: Academic insights and perspectives. Food Engineering Reviews 13(4): 1–36. 10.1007/s12393-020-09273-6

[B25] Liu F Ma ZY Hou LW Diao YZ Wu WP Damm U Song S Cai L (2022) Updating species diversity of *Colletotrichum*, with a phylogenomic overview. Studies in Mycology 101(1): 1–56. 10.3114/sim.2022.101.01 36059896 PMC9365046

[B26] Ma T Yang C Cai F Chen Z (2022) Morpho-cultural, physiological and molecular characterisation of *Colletotrichumnymphaeae* causing anthracnose disease of walnut in China. Microbial Pathogenesis 166: 105537. 10.1016/j.micpath.2022.105537 35430269

[B27] Marin-Felix Y Groenewald JZ Cai L Chen Q Marincowitz S Barnes I Bensch K Braun U Camporesi E Damm U de Beer ZW Dissanayake A Edwards J Giraldo A Hernández-Restrepo M Hyde KD Jayawardena RS Lombard L Luangsa-ard J McTaggart AR Rossman AY Sandoval-Denis M Shen M Shivas RG Tan YP van der Linde EJ Wingfield MJ Wood AR Zhang JQ Zhang Y Crous PW (2017) Genera of phytopathogenic fungi: GOPHY 1. Studies in Mycology 86(1): 99–216. 10.1016/j.simyco.2017.04.002 28663602 PMC5486355

[B28] Mu T Zhang Z Liu R Liu S Li Z Zhang XG Xia JW (2021) Morphological and molecular phylogenetic analyses reveal three species of *Colletotrichum* in Shandong province, China. MycoKeys 85: 57–71. 10.3897/mycokeys.85.75944 34975280 PMC8674231

[B29] O’Donnell K Cigelnik E (1997) Two divergent intragenomic rDNA ITS2 types within monophyletic lineage of the fungus *Fusarium* are nonorthologous. Molecular Phylogenetics and Evolution 7(1): 103–116. 10.1006/mpev.1996.0376 9007025

[B30] Pei D Lu XZ (2011) Walnut germplasm resources in China. China Forestry Press, Beijing.

[B31] Réblová M Miller AN Rossman AY Seifert KA Crous PW Hawksworth DL Abdel-Wahab MA Cannon PF Daranagama DA De Beer ZW Huang SK Hyde KD Jayawardena R Jaklitsch W Gareth Jones EB Ju YM Judith C Maharachchikumbura SSN Pang KL Petrini LE Raja HA Romero AI Shearer C Senanayake IC Voglmayr H Weir BS Wijayawarden NN (2016) Recommendations for competing sexual-asexually typified generic names in *Sordariomycetes* (except *Diaporthales*, *Hypocreales*, and *Magnaporthales*). IMA Fungus 7(1): 131–153. 10.5598/imafungus.2016.07.01.08 27433444 PMC4941682

[B32] Ronquist F Teslenko M van der Mark P Ayres DL Darling A Höhna S Larget B Liu L Suchard MA Huelsenbeck JP (2012) MrBayes 3.2: Efficient bayesian phylogenetic inference and model choice across a large model space. Systematic Biology 61(3): 539–542. 10.1093/sysbio/sys029 22357727 PMC3329765

[B33] Savian L Muniz MFB Poletto T Maculan L Rabuske JE Blume E Sarzi JS (2019) First report of *Colletotrichumnymphaeae* causing anthracnose on *Juglansregia* fruits in southern Brazil. Plant Disease 103(12): 3287. 10.1094/PDIS-06-19-1199-PDN

[B34] Stamatakis A (2014) RAxML Version 8: A tool for phylogenetic analysis and post-analysis of large phylogenies. Bioinformatics (Oxford, England) 30(9): 1312–1313. 10.1093/bioinformatics/btu033 24451623 PMC3998144

[B35] Swofford DL (2002) PAUP*: Phylogenetic analysis using parsimony (*and other methods). Version 4.0b10. Sinauer Associates Sunderland Massachusetts.

[B36] Talhinhas P Baroncelli R (2021) *Colletotrichum* species and complexes: Geographic distribution, host range and conservation status. Fungal Diversity 110(1): 109–198. 10.1007/s13225-021-00491-9

[B37] Tamura K Stecher G Peterson D Filipski A Kumar S (2013) MEGA6: Molecular evolutionary genetics analysis version 6.0. Molecular Biology and Evolution 30(12): 2725–2729. 10.1093/molbev/mst197 24132122 PMC3840312

[B38] Varjas V Kovács CS Lakatos T Tóth T Bujdosó G (2019) First Report of Walnut Anthracnose Caused by *Colletotrichumfioriniae* on English (Persian) Walnut Fruits in Hungary. Plant Disease 103(11): 2964. 10.1094/PDIS-02-19-0286-PDN

[B39] Wang QH Liu XH Fan K Duan CH Niu SG Wu XQ (2016) Identification and biological characteristics of pathogen from *Colletotrichumgloeosporioides.* Shandong Nongye Daxue Xuebao 47: 9–14.

[B40] Wang QH Fan K Li DW Niu SG Hou LQ Wu XQ (2017) Walnut anthracnose caused by *Colletotrichumsiamense* in China. Australasian Plant Pathology 46(6): 585–595. 10.1007/s13313-017-0525-9

[B41] Wang QH Li DW Duan CH Liu XH Niu SG Hou LQ Wu XQ (2018) First report of walnut anthracnose caused by *Colletotrichumfructicola* in China. Plant Disease 102(1): 247. 10.1094/PDIS-06-17-0921-PDN

[B42] Wang XH Liu X Wang R Fa L Chen L Xin XB Zhang YG Tian H Xia MR Hou X (2021) First report of *Colletotrichumaenigma* causing walnut anthracnose in China. Plant Disease 105(1): 225. 10.1094/PDIS-07-20-1430-PDN

[B43] Wang XH Chen L Ma QH Liu X Wang R Fa L Wang HX Hou X Xia MR Yuan W (2023) First report of *Colletotrichumgodetiae* causing anthracnose on walnut (*Juglansregia* and *Juglanssigillata*) in China. Plant Disease 107(8): 2544. 10.1094/PDIS-11-22-2625-PDN

[B44] Wei X Yang SQ Jiang ZG Cui MJ Deng JX Zhang Y (2022) *Colletotrichumjuglandis* sp. nov. (Ascomycota: Glomerellaceae) associated with walnut leaf spot in China. Phytotaxa 556(3): 256–268. 10.11646/phytotaxa.556.3.2

[B45] Weir BS Johnston PR Damm U (2012) The *Colletotrichumgloeosporioides* species complex. Studies in Mycology 73: 115–180. 10.3114/sim0011 23136459 PMC3458417

[B46] White TJ Bruns TD Lee SB Taylor JW (1990) Amplification and direct sequencing of fungal ribosomal RNA genes for phylogenetics. In: Innis MA Gelfand DH Sninsky JJ White TJ (Eds) PCR Protocols: a Guide to Methods and Application.Academic Press, San Diego, CA, USA, 315–322. 10.1016/B978-0-12-372180-8.50042-1

[B47] Yu Z Jiang X Zheng H Zhang H Qiao M (2022) Fourteen new species of foliar *Colletotrichum* associated with the invasive plant *Ageratinaadenophora* and surrounding crops. Journal of Fungi (Basel, Switzerland) 8(2): 185. 10.3390/jof8020185 35205939 PMC8879954

[B48] Zhang L Yin YQ Zhao LL Xie YQ Han J Zhang Y (2023) Two new species of *Colletotrichum* (Glomerellaceae, Glomerellales) causing walnut anthracnose in Beijing. MycoKeys 99: 131–152. 10.3897/mycokeys.99.106812 37719302 PMC10502704

[B49] Zhu YF Yin YF Qu WW Yang KQ (2014) Occurrence and spread of the pathogens on walnut (*Juglansregia*) in Shandong Province, China. Acta Horticulturae (1050): 347–351. 10.17660/ActaHortic.2014.1050.47

[B50] Zhu YZ Liao WJ Zou DX Wu YJ Zhou Y (2015) First report of leaf spot disease on walnut caused by *Colletotrichumfioriniae* in China. Plant Disease 99(2): 289. 10.1094/PDIS-09-14-0938-PDN 30699588

