# Supplementary material for: ﻿Colletotrichum species (Glomerellales, Glomerellaceae) causing walnut anthracnose in China
Source: MycoKeys. 2024 Aug 30;108:95–113. doi: 10.3897/mycokeys.108.127734 (PMC11380052; doi:10.3897/mycokeys.108.127734)
Supplement: Supplementary material 1 — GenBank accession numbers of isolates included in this study [file mycokeys-108-095-s001.docx]

**Supplementary Table S1** GenBank accession numbers of isolates included in this study (newly-generated sequences are in bold).

| Taxa | Strains | Host | Location | GenBank accession number(s) | | | | |
| --- | --- | --- | --- | --- | --- | --- | --- | --- |
|  |  |  |  | ITS | *GAPDH* | *CHS-1* | *ACT* | *TUB2* |
| *Colletotrichum abscissum* | COAD 1877* | *Citrus sinensis* cv. *Pera* | Brazil | KP843126 | KP843129 | KP843132 | KP843141 | KP843135 |
| *C. acerbum* | CBS 128530* | *Malus domestica* | New Zealand | JQ948459 | JQ948790 | JQ949120 | JQ949780 | JQ950110 |
| *C. acutatum* | CBS 112996* | *Carica papaya* | Australia | JQ005776 | JQ948677 | JQ005797 | JQ005839 | JQ005860 |
| *C. aenigma* | ICMP 18608* | *Persea americana* | Israel | JX010244 | JX010044 | JX009774 | JX009443 | JX010389 |
| *C. aeschynomenes* | ICMP 17673* | *Aeschynomene virginica* | USA | JX010176 | JX009930 | JX009799 | JX009483 | JX010392 |
| *C. alatae* | CBS 304.67*, ICMP 17919 | *Dioscorea alata* | India | JX010190 | JX009990 | JX009837 | JX009471 | JX010383 |
| *C. alienum* | ICMP 12071* | *Malus domestica* | New Zealand | JX010251 | JX010028 | JX009882 | JX009572 | JX010411 |
| *C. annellatum* | CBS 129826 | *Hevea indica* | Colombia | JQ005222 | JQ005309 | JQ005396 | JQ005570 | JQ005656 |
| *C. aotearoa* | ICMP 18537* | *Coprosma* sp. | New Zealand | JX010205 | JX010005 | JX009853 | JX009564 | JX010420 |
| *C. arboricola* | CBS 144795*, SAG 53350.12 | *Fuchsia magellanica* | Chile | MH817944 | MH817950 | N/A | MH817956 | MH817962 |
| *C. arecicola* | CGMCC 3.19667* | *Areca catechu* | China | MK914635 | MK935455 | MK935541 | MK935374 | MK935498 |
| *C. artocarpicola* | MFLUCC 18-1167* | *Artocarpus heterophyllus* | Thailand | MN415991 | MN435568 | MN435569 | MN435570 | MN435567 |
| *C. asianum* | ICMP 18580* | *Coffea arabica* | Thailand | FJ972612 | JX010053 | JX009867 | JX009584 | JX010406 |
| *C. australe* | CBS 116478* | *Trachycarpus fortunei* | South Africa | JQ948455 | JQ948786 | JQ949116 | JQ949776 | JQ950106 |
| *C. australianum* | VPRI 43075* | *Citrus sinensis* | Australia | MG572138 | MG572127 | MW091987 | MN442109 | MG572149 |
| *C. beeveri* | CBS 128527* | *Brachyglottis repanda* | New Zealand | JQ005171 | JQ005258 | JQ005345 | JQ005519 | JQ005605 |
| ***C. boninense*** | **FY-10.2** | ***Juglans regia*** | **China** | **OR287136** | **OR295515** | **OR295471** | **OR295493** | **OR295537** |
| ***C. boninense*** | **BSY-1.2** | ***Juglans regia*** | **China** | **OR594147** | **OR608467** | **OR608467** | **OR608458** | **OR916417** |
| *C. boninense* | CBS 123756 | *Crinum asiaticum* var. *sinicum* | Japan | JQ005154 | JQ005241 | JQ005328 | JQ005502 | JQ005589 |
| *C. boninense* | MAFF 305972*, CBS 123755 | *Crinum asiaticum* var. *sinicum* | Japan | JQ005153 | JQ005240 | JQ005327 | JQ005501 | JQ005588 |
| *C. boninense* | CBS 128526 | *Dacrycarpus dacrydioides* | New Zealand | JQ005162 | JQ005249 | JQ005336 | JQ005510 | JQ005596 |
| *C. brasiliense* | CBS 125801* | *Passiflora edulis* | Brazil | JQ005235 | JQ005322 | JQ005409 | JQ005583 | JQ005669 |
| *C. brassicicola* | CBS 101059* | *Brassica oleracea* var. *gemmifera* | New Zealand | JQ005172 | JQ005259 | JQ005346 | JQ005520 | JQ005606 |
| *C. brisbanense* | CBS 292.67* | *Capsicum annuum* | Australia | JQ948291 | JQ948621 | JQ948952 | JQ949612 | JQ949942 |
| *C. bromeliacearum* | LC0951* | *Bromeliad* | China | MZ595832 | MZ664077 | MZ799267 | MZ664130 | MZ673956 |
| *C. cairnsense* | BRIP 63642* | *Capsicum annuum* | Australia | KU923672 | KU923704 | KU923710 | KU923716 | KU923688 |
| *C. camelliae* | CGMCC 3.14925, LC1364* | *Camellia sinensis* | China | KJ955081 | KJ954782 | MZ799255 | KJ954363 | KJ955230 |
| *C. camelliae-japonicae* | CGMCC 3.18118* | *Camellia japonica* | China | KX853165 | KX893584 | MZ799271 | KX893576 | KX893580 |
| *C. carthami* | SAPA 100011* | *Carthamus tinctorium* | Japan | AB696998 | N/A | N/A | N/A | AB696992 |
| *C. catinaense* | CBS 142417*, CPC 27978 | *Citrus reticulata* | Italy | KY856400 | KY856224 | KY856136 | KY855971 | KY856482 |
| *C. chamaedoreae* | NN052885* | *Chamaedorea erumpens* | China | MZ595890 | MZ664084 | MZ799274 | MZ664188 | MZ674008 |
| *C. changpingense* | MFLUCC 15-0022, CGMCC 3.17582* | Rhizome of *Fragaria × ananass* | China | KP683152 | MZ664048 | KP852449 | KP683093 | MZ673952 |
| *C. chiangmaiense* | MFLUCC 18-0945* | *Magnolia garrettii* | Thailand | MW346499 | MW548592 | MW623653 | MW655578 | N/A |
| ***C. chinensis*** | **JF715-6.1** | ***Juglans regia*** | **China** | **OR287151** | **OR295533** | **OR295489** | **OR295511** | **OR295555** |
| ***C. chinensis*** | **JF715-6.3** | ***Juglans regia*** | **China** | **OR287152** | **OR295534** | **OR295490** | **OR295512** | **OR295556** |
| ***C. chinensis*** | **QCG-1.1** | ***Juglans regia*** | **China** | **OR287154** | **OR295535** | **OR295491** | **OR295513** | **OR295557** |
| ***C. chinensis*** | **QCG-1.3** | ***Juglans regia*** | **China** | **OR287156** | **OR295536** | **OR295492** | **OR295514** | **OR295558** |
| *C. chongqingense* | CB0612* | *Camellia sinensis* | China | MG602060 | MG602022 | MT976117 | MG602044 | N/A |
| *C. chrysanthemi* | IMI 364540 | *Chrysanthemum coronarium* | China | JQ948273 | JQ948603 | JQ948934 | JQ949594 | JQ949924 |
| *C. chrysophilum* | CMM 4268* | *Musa* sp. | Brazil | KX094252 | KX094183 | KX094083 | KX093982 | KX094285 |
| *C. cigarro* | ICMP 18539* | *Olea europaea* | Australia | JX010230 | JX009966 | JX009800 | JX009523 | JX010434 |
| *C. citricola* | CBS 134228* | *Citrus unshiu* | China | KC293576 | KC293736 | N/A | KC293616 | KC293656 |
| ***C. citrulli*** | **YJZY-3.3** | ***Juglans regia*** | **China** | **OR287135** | **OR295516** | **OR295472** | **OR295494** | **OR295538** |
| ***C. citrulli*** | **YJZY-3.2** | ***Juglans regia*** | **China** | **OR594146** | **OR608477** | **OR608468** | **OR608459** | **OR916418** |
| *C. citrulli* | CAASZT54 | *Citrullus lanatus* | China | MZ475134 | OL456686 | OL901154 | OL449284 | OL456645 |
| *C. citrulli* | CAASZT52 | *Citrullus lanatus* | China | MZ475133 | OL456685 | OL901153 | OL449283 | OL456644 |
| *C. clidemiae* | ICMP 18658* | *Clidemia hirta* | USA, Hawaii | JX010265 | JX009989 | JX009877 | JX009537 | JX010438 |
| *C. cobbittiense* | BRIP 66219* | *Cordyline stricta × C. australis* | Australia | MH087016 | MH094133 | MH094135 | MH094134 | MH094137 |
| *C. colombiense* | CBS 129818* | *Passiflora edulis* | Colombia | JQ005174 | JQ005261 | JQ005348 | JQ005522 | JQ005608 |
| *C. condaoense* | CBS 134299* | *Ipomoea pes-caprae* | Vietnam | MH229914 | MH229920 | MH229926 | N/A | MH229923 |
| *C. conoides* | CGMCC 3.17615 | *Chili pepper* | China | KP890168 | KP890162 | KP890156 | KP890144 | KP890174 |
| *C. constrictum* | CBS 128504* | *Citrus limon* | New Zealand | JQ005238 | JQ005325 | JQ005412 | JQ005586 | JQ005672 |
| *C. cordylinicola* | ICMP 18579* | *Cordyline fruticosa* | Thailand | JX010226 | JX009975 | JX009864 | HM470235 | JX010440 |
| *C. cosmi* | CBS 853.73* | *Cosmos* sp. | Netherlands | JQ948274 | JQ948604 | JQ948935 | JQ949595 | JQ949925 |
| *C. costaricense* | CBS 330.75* | *Coffea arabica* cv. *Typica, berry* | Costa Rica | JQ948180 | JQ948510 | JQ948841 | JQ949501 | JQ949831 |
| *C. cuscutae* | IMI 304802* | *Cuscuta* sp. | Dominica | JQ948195 | JQ948525 | JQ948856 | JQ949516 | JQ949846 |
| *C. cymbidiicola* | IMI 347923* | *Cymbidium* sp. | Australia | JQ005166 | JQ005253 | JQ005340 | JQ005514 | JQ005600 |
| *C. dacrycarpi* | CBS 130241* | *Dacrycarpus dacrydioides* | New Zealand | JQ005236 | JQ005323 | JQ005410 | JQ005584 | JQ005670 |
| *C. dimorphum* | CGMCC 3.16083* | *Ageratina adenophora* | China | OK030867 | OK513670 | OK513566 | OK513606 | OK513636 |
| *C. dimorphum* | YMF 1.07303 | *Ageratina adenophora* | China | OK030866 | OK513669 | OK513565 | OK513605 | OK513635 |
| *C. diversum* | LC11292* | *Philodendron selloum* | China | MZ595844 | MZ664081 | MZ799272 | MZ664142 | MZ673965 |
| *C. doitungense* | MFLUCC 14-0128* | *Dendrobium* sp. | Thailand | MF448524 | MH049480 | N/A | MH376385 | MH351277 |
| *C. dracaenigenum* | MFLUCC 19-0430* | *Dracaena* sp. | Thailand | MN921250 | MT215577 | MT215575 | MT313686 | N/A |
| *C. endophyticum* | MFLUCC 13-0418*, LC0324 | *Pennisetum purpureum* | Thailand | KC633854 | KC832854 | MZ799261 | KF306258 | MZ673954 |
| *C. eriobotryae* | GLMC 1935*, Cer001 | *Eriobotrya japonica* | Taiwan | MF772487 | MF795423 | MN191653 | MN191648 | MF795428 |
| *C. feijoicola* | CBS 144633* | *Acca sellowiana* | Portugal | MK876413 | MK876475 | N/A | MK876466 | MK876507 |
| *C. fici-septicae* | MFLU 19-27708* | *Capsicum annuum* | China | KP145441 | KP145413 | KP145385 | KP145329 | KP145469 |
| *C. fioriniae* | IMI 363003 | *Camellia reticulata* | China | JQ948339 | JQ948669 | JQ949000 | JQ949660 | JQ949990 |
| *C. fioriniae* | CBS 128517* | *Fiorinia externa* | USA | JQ948292 | JQ948622 | JQ948953 | JQ949613 | JQ949943 |
| *C. fioriniae* | CBS 129948 | *Tulipa* sp. | UK | JQ948344 | JQ948674 | JQ949005 | JQ949665 | JQ949995 |
| *C. fioriniae* | CBS 119293 | *Vaccinium corymbosum* | New Zealand | JQ948314 | JQ948644 | JQ948975 | JQ949635 | JQ949965 |
| ***C. fioriniae*** | **WX-1.1** | ***Juglans regia*** | **China** | **OR287137** | **OR295517** | **OR295473** | **OR295495** | **OR295539** |
| ***C. fioriniae*** | **XL-16.3** | ***Juglans regia*** | **China** | **OR287139** | **OR295518** | **OR295474** | **OR295496** | **OR295540** |
| *C. fructicola* | ICMP 18581* | *Coffea arabica* | Thailand | JX010165 | JX010033 | JX009866 | FJ907426 | JX010405 |
| *C. fructicola* | ICMP 18646 | *Tetragastris panamensis* | Panama | JX010173 | JX010032 | JX009874 | JX009581 | JX010409 |
| ***C. fructicola*** | **BSG-9.1** | ***Juglans regia*** | **China** | **OR287138** | **OR295519** | **OR295475** | **OR295497** | **OR295541** |
| ***C. fructicola*** | **BSY-2.1** | ***Juglans regia*** | **China** | **OR594148** | **OR608478** | **OR608469** | **OR608460** | **OR916419** |
| *C. fructivorum* | CBS 133125* | *Vaccinium macrocarpon* | Burlington | JX145145 | MZ664047 | MZ799259 | MZ664126 | JX145196 |
| *C. gloeosporioides* | IMI 356878*, ICMP 17821, CBS 112999 | *Citrus sinensis* | Italy | JX010152 | JX010056 | JX009818 | JX009531 | JX010445 |
| *C. gloeosporioides* | CBS 273.51(*), ICMP 19121 | *Citrus limon* | Italy | JX010148 | JX010054 | JX009903 | JX009558 | N/A |
| *C. gloeosporioides* | DAR 76936, ICMP 18738 | *Carya illinoinensis* | Australia | JX010151 | JX009976 | JX009797 | JX009542 | N/A |
| *C. gloeosporioides* | ICMP12939 | *Citrus* sp. | New Zealand | JX010149 | JX009931 | JX009747 | JX009462 | N/A |
| *C. gloeosporioides* | CBS 119204, ICMP 18678 | *Pueraria lobata* | USA | JX010150 | JX010013 | JX009790 | JX009502 | N/A |
| *C. godetiae* | CBS 133.44* | *Clarkia hybrida ,* cv. *Kelvon Glory* | Denmark | JQ948402 | JQ948733 | JQ949063 | JQ949723 | JQ950053 |
| *C. godetiae* | CBS 126522 | *Prunus cerasus* | Netherlands | JQ948411 | JQ948742 | JQ949072 | JQ949732 | JQ950062 |
| ***C. godetiae*** | **FY-23.1** | ***Juglans regia*** | **China** | **OR287141** | **OR295520** | **OR295476** | **OR295498** | **OR295542** |
| ***C. godetiae*** | **FY-12.2** | ***Juglans regia*** | **China** | **OR594151** | **OR608479** | **OR608470** | **OR608461** | **OR916420** |
| *C. grevilleae* | CBS 132879* | *Grevillea* sp. | Italy | KC297078 | KC297010 | KC296987 | KC296941 | KC297102 |
| *C. grossum* | CGMCC 3.17614*, CAUG7 | *Chili pepper* | China | KP890165 | KP890159 | KP890153 | KP890141 | KP890171 |
| *C. guajavae* | IMI350839* | *Psidium guajava* | India | JQ948270 | JQ948600 | JQ948931 | JQ949591 | JQ949921 |
| *C. hebeiense* | MFLUCC 13-0726* | *Vitis vinifera* | China | KF156863 | KF377495 | KF289008 | KF377532 | KF288975 |
| *C. hederiicola* | MFLU 15-0689* | *Hedera helix* | Italy | MN631384 | N/A | MN635794 | MN635795 | N/A |
| *C. helleniense* | CBS 142418*, CPC 26844 | *Poncirus trifoliata* | Greece, Arta | KY856446 | KY856270 | KY856186 | KY856019 | KY856528 |
| *C. henanense* | CGMCC 3.17354* | *Camellia sinensis* | China | KJ955109 | KJ954810 | MZ799256 | KM023257 | KJ955257 |
| *C. hippeastri* | CBS 125376* | *Hippeastrum vittatum* | China | JQ005231 | JQ005318 | JQ005405 | JQ005579 | JQ005665 |
| *C. horii* | NBRC 7478* | *Diospyros kaki* | Japan | GQ329690 | GQ329681 | JX009752 | JX009438 | JX010450 |
| *C. hystricis* | CBS 142411* | *Citrus hystrix* | Italy, Catania | KY856450 | KY856274 | KY856190 | KY856023 | KY856532 |
| *C. indonesiense* | CBS 127551* | *Eucalyptus* sp. | Indonesia | JQ948288 | JQ948618 | JQ948949 | JQ949609 | JQ949939 |
| *C. javanense* | CBS 144963* | *Capsicum annuum* | Indonesia | MH846576 | MH846572 | MH846573 | MH846575 | MH846574 |
| *C. jiangxiense* | CGMCC 3.17361*, LF488 | *Camellia sinensis* | China | KJ955149 | KJ954850 | MZ799257 | KJ954427 | OK236389 |
| *C. johnstonii* | CBS 128532* | *Solanum lycopersicum* | New Zealand | JQ948444 | JQ948774 | JQ949105 | JQ949765 | JQ950095 |
| *C. juglandicola* | CGMCC3.24312* | *Juglans regia* | China | OQ263015 | OQ282973 | OR004793 | OQ282966 | OQ282980 |
| *C. juglandicola* | CGMCC3.24313 | *Juglans regia* | China | OQ263018 | OQ282977 | OR004797 | OQ282970 | OQ282984 |
| ***C. juglandicola*** | **CJLG-11.3** | ***Juglans regia*** | **China** | **OR287149** | **OR295521** | **OR295477** | **OR295499** | **OR295543** |
| ***C. juglandicola*** | **DJKG-2.3** | ***Juglans regia*** | **China** | **OR287147** | **OR295522** | **OR295478** | **OR295500** | **OR295544** |
| *C. juglandis* | YZU191277* | *Juglans regia* | China | OM755762 | OM913576 | OM913546 | OM913553 | N/A |
| *C. juglandis* | YZU201192 | *Juglans regia* | China | OM755764 | OM913578 | OM913548 | OM913555 | N/A |
| *C. kahawae* | IMI 319418*, ICMP 17816 | *Coffea arabica* | Kenya | JX010231 | JX010012 | JX009813 | JX009452 | JX010444 |
| *C. karstii* | CBS 106.91 | *Carica papaya* | Brazil | JQ005220 | JQ005307 | JQ005394 | JQ005568 | JQ005654 |
| *C. karstii* | CBS 861.72 | *Bombax aquaticum* | Brazil | JQ005184 | JQ005271 | JQ005358 | JQ005532 | JQ005618 |
| *C. karstii* | CBS 110779 | *Eucalyptus grandis* | South Africa | JQ005198 | JQ005285 | JQ005372 | JQ005546 | JQ005632 |
| ***C. karstii*** | **FY-2.2** | ***Juglans regia*** | **China** | **OR287140** | **OR295523** | **OR295479** | **OR295501** | **OR295545** |
| ***C. karstii*** | **FYY-11.2** | ***Juglans regia*** | **China** | **OR594149** | **OR608480** | **OR608471** | **OR608462** | **OR916421** |
| *C. kinghornii* | CBS 198.35* | *Phormium* sp. | UK | JQ948454 | JQ948784 | JQ949115 | JQ949775 | JQ950105 |
| *C. kniphofiae* | CBS 143496* | *Kniphofia uvaria* | UK | MH107884 | MH107998 | MH107990 | MH107975 | MH108037 |
| *C. laticiphilum* | CBS 112989* | *Hevea brasiliensis* | India | JQ948289 | JQ948619 | JQ948950 | JQ949610 | JQ949940 |
| *C. lauri* | MFLUCC:17-0205* | *Laurus nobilis* | Italy | KY514347 | KY514344 | KY514341 | KY514338 | KY514350 |
| *C. ledongense* | CGMCC3.18888*, LD1680 | *Quercus palustris* | China | MG242008 | MG242016 | MG242018 | MG242014 | MG242010 |
| *C. limetticola* | CBS 114.14* | *Citrus aurantifolia* | USA, Florida | JQ948193 | JQ948523 | JQ948854 | JQ949514 | JQ949844 |
| *C. limonicola* | CBS 142410* | *Citrus limon* | Malta, Gozo | KY856472 | KY856296 | KY856213 | KY856045 | KY856554 |
| *C. lupini* | CBS 109225* | *Lupinus albus* | Ukraine | JQ948155 | JQ948485 | JQ948816 | JQ949476 | JQ949806 |
| *C. makassarense* | CBS 143664* | *Capsicum annuum* | Indonesia | MH728812 | MH728820 | MH805850 | MH781480 | MH846563 |
| *C. melonis* | CBS 159.84* | *Cucumis melo* | Brazil | JQ948194 | JQ948524 | JQ948855 | JQ949515 | JQ949845 |
| *C. mengyinense* | SAUCC0702* | *Rosa chinensis* | China | MW786742 | MW846240 | MW883686 | MW883695 | MW888970 |
| *C. mengyinense* | SAUCC200912 | *Juglans regia* | China | MW786689 | MW876472 | MW883687 | MW883696 | MW888971 |
| ***C. mengyinense*** | **XL-7.1** | ***Juglans regia*** | **China** | **OR291150** | **OR295524** | **OR295485** | **OR295502** | **OR295546** |
| ***C. mengyinense*** | **YJZG-14.2** | ***Juglans regia*** | **China** | **OR290968** | **OR295525** | **OR295486** | **OR295503** | **OR295547** |
| ***C. mengyinense*** | **YJZY-10.1** | ***Juglans regia*** | **China** | **OR291152** | **OR295526** | **OR295487** | **OR295504** | **OR295548** |
| ***C. mengyinense*** | **CJLY-9.2** | ***Juglans regia*** | **China** | **OR594150** | **OR608483** | **OR608474** | **OR608465** | **OR916424** |
| *C. miaoliense* | NTUCC 20-001-1*, ML1040 | *Fragaria × ananassa* | Taiwan | MK908419 | MK908470 | MK908522 | MK908573 | MK908624 |
| *C. musae* | CBS 116870* | *Musa* sp. | USA | JX010146 | JX010050 | JX009896 | JX009433 | HQ596280 |
| *C. nanhuaensis* | CGMCC 3.18962* | *Ageratina adenophora* | China | OK030870 | OK513673 | OK513569 | OK513609 | OK513639 |
| *C. nanhuaensis* | YMF 1.04990 | *Ageratina adenophora* | China | OK030871 | OK513674 | OK513570 | OK513610 | OK513640 |
| *C. novae-zelandiae* | CBS 128505* | *Capsicum annuum* | New Zealand | JQ005228 | JQ005315 | JQ005402 | JQ005576 | JQ005662 |
| *C. nupharicola* | CBS 470.96* | *Nuphar lutea subsp. polysepala* | USA | JX010187 | JX009972 | JX009835 | JX009437 | JX010398 |
| *C. nymphaeae* | CBS 515.78* | *Nymphaea alba* | Netherlands | JQ948197 | JQ948527 | JQ948858 | JQ949518 | JQ949848 |
| *C. nymphaeae ‘C. citri’* | CBS 134233, YN01 | *Citrus aurantifolia* | China | KC293581 | KC293741 | KY856138 | KY855973 | KC293661 |
| *C. oncidii* | CBS 129828* | *Oncidium* sp. | Germany | JQ005169 | JQ005256 | JQ005343 | JQ005517 | JQ005603 |
| *C. pandanicola* | MFLUCC 17-0571* | *Pandanaceae* | Thailand | MG646967 | MG646934 | MG646931 | MG646938 | MG646926 |
| ***C. pandanicola*** | **JF715-2.1** | ***Juglans regia*** | **China** | **OR287143** | **OR295527** | **OR295480** | **OR295505** | **OR295549** |
| ***C. pandanicola*** | **XL-3.2** | ***Juglans regia*** | **China** | **OR287142** | **OR295528** | **OR295481** | **OR295506** | **OR295550** |
| ***C. pandanicola*** | **XL-9.3** | ***Juglans regia*** | **China** | **OR287146** | **OR295529** | **OR295482** | **OR295507** | **OR295551** |
| ***C. pandanicola*** | **CJLG-10.2** | ***Juglans regia*** | **China** | **OR594152** | **OR608481** | **OR608472** | **OR608463** | **OR916422** |
| *C. paranaense* | CBS 134729*, CPC20901 | *Malus domestica* | Paraná | KC204992 | KC205026 | KC205043 | KC205077 | KC205060 |
| *C. parsonsiae* | CBS 128525* | *Parsonsia capsularis endophyte* | New Zealand | JQ005233 | JQ005320 | JQ005407 | JQ005581 | JQ005667 |
| *C. paxtonii* | IMI 165753* | *Musa* sp. | Saint Lucia | JQ948285 | JQ948615 | JQ948946 | JQ949606 | JQ949936 |
| *C. peakense* | CGMCC3.24308* | *Juglans regia* | China | OQ263017 | OQ282975 | OR004795 | OQ282968 | OQ282982 |
| *C. peakense* | CGMCC3.24307 | *Juglans regia* | China | OQ263016 | OQ282974 | OR004794 | OQ282967 | OQ282981 |
| ***C. penkense*** | **SSL715-1.3** | ***Juglans regia*** | **China** | **OR287148** | **OR295530** | **OR295488** | **OR295508** | **OR295552** |
| ***C. penkense*** | **SSL715-3.2** | ***Juglans regia*** | **China** | **OR594154** | **OR608484** | **OR608475** | **OR608466** | **OR916425** |
| *C. perseae* | CBS 141365* | *Avocado* | Israel | KX620308 | KX620242 | MZ799260 | KX620145 | KX620341 |
| *C. petchii* | CBS 378.94* | *Dracaena marginata* | Italy | JQ005223 | JQ005310 | JQ005397 | JQ005571 | JQ005657 |
| *C. phormii* | CBS 118194* | *Phormium* sp. | Germany | JQ948446 | JQ948777 | JQ949107 | JQ949767 | JQ950097 |
| *C. phyllanthi* | CBS 175.67* | *Phyllanthus acidus* | India | JQ005221 | JQ005308 | JQ005395 | JQ005569 | JQ005655 |
| *C.populi* | HMBFU191 | *Poplar* | China | AB632347 | JN211081 | N/A | JN184704 | JN862898 |
| *C. proteae* | CBS 132882* | *Protea* sp. | South Africa | KC297079 | KC297009 | KC296986 | KC296940 | KC297101 |
| *C. pseudoactatum* | CBS 436.77 | *Pinus radiata* | Chile | JQ948480 | JQ948811 | JQ949141 | JQ949801 | JQ950131 |
| *C. pseudotheobromicola* | MFLUCC 18-1602* | *Prunus avium* | China | MH817395 | MH853675 | MH853678 | MH853681 | MH853684 |
| *C. psidii* | CBS 145.29*, ICMP 19120 | *Psidium* sp. | Italy | JX010219 | JX009967 | JX009901 | JX009515 | JX010443 |
| *C. pyricola* | CBS 128531* | *Pyrus communis* | New Zealand | JQ948445 | JQ948776 | JQ949106 | JQ949766 | JQ950096 |
| *C. queenslandicum* | ICMP 1778* | *Carica papaya* | Australia | JX010276 | JX009934 | JX009899 | JX009447 | JX010414 |
| *C. rhexiae* | CBS 133134* | *Rhexia virginica* | Sussex | JX145128 | MZ664046 | MZ799258 | MZ664127 | JX145179 |
| *C. rhombiforme* | CBS 129953* | *Olea europaea* | Portugal | JQ948457 | JQ948788 | JQ949118 | JQ949778 | JQ950108 |
| *C. roseum* | CBS 145754* | *Lapageria rosea* | Chile | MK903611 | MK903603 | N/A | MK903604 | MK903607 |
| *C. salicis* | CBS 607.94* | *Salix* sp. | Netherlands | JQ948460 | JQ948791 | JQ949121 | JQ949781 | JQ950111 |
| *C. salsolae* | ICMP 19051* | *Salsola tragus* | Hungary | JX010242 | JX009916 | JX009863 | JX009562 | JX010403 |
| *C. schimae* | NN046984*, LC13880 | *Schima* sp. | China | MZ595885 | MZ664105 | MZ799347 | MZ664183 | MZ674003 |
| *C. scovillei* | CBS 126529* | *Capsicum* sp. | Indonesia | JQ948267 | JQ948597 | JQ948928 | JQ949588 | JQ949918 |
| *C. siamense* | ICMP 18578* | *Coffea arabica* | Thailand | JX010171 | JX009924 | JX009865 | FJ907423 | JX010404 |
| *C. siamense* | ICMP 18575 | *Capsicum annuum* | Thailand | JX010256 | JX010059 | JX009785 | JX009455 | N/A |
| *C. siamense* | CBS 125378 | *Hymenocallis americana* | China | JX010278 | JX010019 | GQ856730 | GQ856775 | JX010410 |
| *C. siamense* | CBS 130420 | *Jasminum sambac* | Vietnam | HM131511 | HM131497 | JX009895 | HM131507 | JX010415 |
| ***C. siamense*** | **XL-4.3** | ***Juglans regia*** | **China** | **OR287145** | **OR295531** | **OR295483** | **OR295509** | **OR295553** |
| ***C. siamense*** | **XL-5.1** | ***Juglans regia*** | **China** | **OR287144** | **OR295532** | **OR295484** | **OR295510** | **OR295554** |
| ***C. siamense*** | **QCG-26.2** | ***Juglans regia*** | **China** | **OR594153** | **OR608482** | **OR608473** | **OR608464** | **OR916423** |
| *C. simmondsii* | CBS 122122* | *Carica papaya* | Australia | JQ948276 | JQ948606 | JQ948937 | JQ949597 | JQ949927 |
| *C. sloanei* | IMI 364297* | *Theobroma cacao* | Malaysia | JQ948287 | JQ948617 | JQ948948 | JQ949608 | JQ949938 |
| *C. subsalicis* | LC13863*, CQ1168 | *Populus alba* | China | MZ852849 | N/A | MZ799346 | MZ664128 | MZ673953 |
| *C. syzygiicola* | MFLUCC 10-0624* | *Syzygium samarangense* | Thailand | KF242094 | KF242156 | N/A | KF157801 | KF254880 |
| *C. tainanense* | CBS 143666* | *Capsicum annuum* | Taiwan | MH728818 | MH728823 | MH805845 | MH781475 | MH846558 |
| *C. tamarilloi* | CBS 129814* | *Solanum betaceum* | Colombia | JQ948184 | JQ948514 | JQ948845 | JQ949505 | JQ949835 |
| *C. temperatum* | CBS 133122* | *Vaccinium macrocarpon* | Bronx | JX145159 | MZ664045 | MZ799254 | MZ664125 | JX145211 |
| *C. tengchongense* | YMF 1.04950 | *Isoetes sinensis* | China | OL842169 | OL981264 | OL981290 | OL981238 | N/A |
| *C. ti* | ICMP 4832* | *Cordyline* sp. | New Zealand | JX010269 | JX009952 | JX009898 | JX009520 | JX010442 |
| *C. torulosum* | CBS 128544* | *Solanum melongena* | New Zealand | JQ005164 | JQ005251 | JQ005338 | JQ005512 | JQ005598 |
| *C. tropicale* | CBS 124949* | *Theobroma cacao* | Panama | JX010264 | JX010007 | JX009870 | JX009489 | JX010407 |
| *C. viniferum* | GZAAS 5.08601*, yg1 | *Vitis vinifera* cv. *Shuijing* | China | JN412804 | JN412798 | N/A | JN412795 | N/A |
| *C. vulgaris* | YMF 1.04940 | *Hippuris vulgaris* | China | OL842170 | OL981265 | OL981291 | OL981239 | N/A |
| *C. walleri* | CBS 125472* | *Coffea* sp. | Vietnam | JQ948275 | JQ948605 | JQ948936 | JQ949596 | JQ949926 |
| *C. wanningense* | CGMCC 3.18936*, Hainan14 | Rubber tree | China | MG830462 | MG830318 | MG830302 | MG830270 | MG830286 |
| *C. watphraense* | MFLUCC 14-0123* | *Dendrobium* sp. | Thailand | MF448523 | MH049479 | N/A | MH376384 | MH351276 |
| *C. wuxiense* | CGMCC 3.17894* | *Camellia sinensis* | China | KU251591 | KU252045 | KU251939 | KU251672 | KU252200 |
| *C. xanthorrhoeae* | BRIP 45094* | *Xanthorrhoea preissii* | Australia | JX010261 | JX009927 | JX009823 | JX009478 | JX010448 |
| *C. xishuangbannaense* | MFLUCC 19-0107* | *Magnolia liliifera* | China | MW346469 | MW537586 | MW660832 | MW652294 | N/A |
| *C. yulongense* | CFCC 50818* | *Vaccinium dunalianum* var. *urophyllum* | China | MH751507 | MK108986 | MH793605 | MH777394 | MK108987 |
| *C. yunanjiangensis* | CGMCC 3.18964* | *Ageratina adenophora* | China | OK030885 | OK513686 | OK513583 | OK513620 | OK513649 |
| *C.theobromicola* | CBS 124945, ICMP 18649* | *Theobroma cacao* | Panama | JX010294 | JX010006 | JX009869 | JX009444 | JX010447 |
| *Colletotrichum* sp. | CBS 123921 | *Dendrobium kingianum* | Japan | JQ005163 | JQ005250 | JQ005337 | JQ005511 | JQ005597 |

* = ex-type or authentic culture, (*) = ex-type or authentic culture of synonymised taxon. Sequences downloaded from GenBank, not generated as part of this project are in bold font.
