# Supplementary material for: ﻿Colletotrichum species (Glomerellales, Glomerellaceae) causing walnut anthracnose in China
Source: MycoKeys. 2024 Aug 30;108:95–113. doi: 10.3897/mycokeys.108.127734 (PMC11380052; doi:10.3897/mycokeys.108.127734)
Supplement: Supplementary material 2 — The occurrence of 12 Colletotrichum species [file mycokeys-108-095-s002.docx]

**Supplementary Table S2** The occurrence of 12 *Colletotrichum* species.

| Species | Occurrence on walnut/Sample (Isolation rate) | Country/Location |
| --- | --- | --- |
| *Colletotrichum boninense* | 2/127 (1.2%) | Yunnan |
| *C. chinensis* | 23/169 (13.9%) | Beijing, Shandong |
| *C. citrulli* | 2/55 (1.2%) | Shandong |
| *C. fioriniae* | 4/93 (2.4%) | Gansu, Shaanxi |
| *C. fructicola* | 6/127 (3.6%) | Yunnan |
| *C. godetiae* | 9/138 (5.5%) | Shaanxi, Yunnan |
| *C. juglandicola* | 17/114 (10.3%) | Beijing, Shandong |
| *C. karstii* | 2/127 (1.2%) | Yunnan |
| *C. mengyinense* | 40/233 (24.2%) | Gansu, Shandong, Shaanxi |
| *C. pandanicola* | 17/169 (10.3%) | Beijing, Shandong, Shaanxi |
| *C. peakense* | 7/55 (4.2%) | Beijing |
| *C. siamense* | 36/271 (21.8%) | Beijing, Shandong, Shaanxi, Sichuan, Xinjiang |
| Total | 165/470 | Beijing, Gansu, Shandong, Shaanxi, Sichuan, Xinjiang, Yunnan |
