# Supplementary material for: ﻿Colletotrichum species (Glomerellales, Glomerellaceae) causing walnut anthracnose in China
Source: MycoKeys. 2024 Aug 30;108:95–113. doi: 10.3897/mycokeys.108.127734 (PMC11380052; doi:10.3897/mycokeys.108.127734)
Supplement: Supplementary material 3 — Morphological comparisons of twelve Colletotrichum species in this study and their hosts in previously reported [file mycokeys-108-095-s003.docx]

**Supplementary Table S3** Morphological comparisons of twelve *Colletotrichum* species in this study and their hosts in previously reported.

| Species | Conidial size | Hosts | Reference(s) |
| --- | --- | --- | --- |
| *Colletotrichum boninense* | NO | Reported on 18 family plants, ***Juglans regia*** | Talhinhas and Baroncelli, 2023, **this study (new record)** |
| *C. chinensis* | 16.4 ± 1.0 × 5.0 ± 0.3 | ***Juglans regia*** | **this study (novel species)** |
| *C. citrulli* | 16.2 ± 1.3 × 5.6 ± 0.4 | *Citrullus lanatus*， ***Juglans regia*** | Guo et al. 2022, **this study (new record)** |
| *C. fioriniae* | 13.9 ± 0.9 × 3.9 ± 0.3 | Reported on 37 family plants, ***Juglans regia*** | Talhinhas and Baroncelli, 2023, **this study** |
| *C. fructicola* | 15.7 ± 0.9 × 5.4 ± 0.3 | Reported on 55 family plants, ***Juglans regia*** | Talhinhas and Baroncelli, 2023, **this study** |
| *C. godetiae* | 15.2 ± 0.9 × 4.7 ± 0.3 | Reported on 18 family plants, ***Juglans regia*** | Talhinhas and Baroncelli, 2023, Wang et al. 2023, **this study** |
| *C. juglandicola* | 17.2 ± 2.4 × 6.0 ± 0.5 | ***Juglans regia*** | Zhang et al. 2023, **this study** |
| *C. karsti* | 16.1 ± 1.6 × 6.0 ± 0.4 | Reported on 47 family plants, ***Juglans regia*** | Talhinhas and Baroncelli, 2023, **this study (new record)** |
| *C. mengyinense* | 15.4 ± 1.0 × 5.6 ± 0.4 | *Rosachinensis*, ***Juglans regia*** | Mu et al. 2021, **this study** |
| *C. pandanicola* | 14.8 ± 0.8 × 5.1 ± 0.4 | ***Juglans regia****,* *Pandanus* sp. and *Fragaria* × *ananassa* Duch. | Tibpromma et al. 2018, Mu et al. 2021, Yan et al. 2023, **this study** |
| *C. peakense* | 17.7± 0.9 × 5.1± 0.3 | ***Juglans regia*** | Zhang et al. 2023, **this study** |
| *C. siamense* | 14.5± 0.9 × 5.2± 0.4 | Reported on 65 family plants, ***Juglans regia*** | Talhinhas and Baroncelli, 2023, Wang et al. 2016, 2017, **this study** |

Notes: NO = No conidia observed.
