# Supplementary material for: ﻿Colletotrichum species (Glomerellales, Glomerellaceae) causing walnut anthracnose in China
Source: MycoKeys. 2024 Aug 30;108:95–113. doi: 10.3897/mycokeys.108.127734 (PMC11380052; doi:10.3897/mycokeys.108.127734)
Supplement: Supplementary material 4 — Mycelial diameter of twelve Colletotrichum species and lesion lengths formed on walnut fruits and leaves [file mycokeys-108-095-s004.docx]

**Supplementary Table S4** Mycelial diameter of twelve *Colletotrichum* species and lesion lengths formed on walnut fruits and leaves.

| Species | Mycelial growth diameter on PDA ^Z^ | Mycelial growth diameter on MEA ^Z^ | Lesion length on fruits (mm) ^Z^ | Lesion length on leaves (mm) ^Z^ |
| --- | --- | --- | --- | --- |
| *Colletotrichum boninense* | 63.4 ± 0.9 d | 64.0 ± 2.4 e | 8.4 ± 1.0 bc | 11.8 ± 4.6 cd |
| *C. chinensis* | 80.0 ± 0 a | 75.7 ± 0.6 abc | 6.5 ± 3.1 cd | 11.2 ± 4.0 cd |
| *C. citrulli* | 66.1 ± 0.9 d | 78.8 ± 6.3 a | 6.6 ± 2.1 cd | 20.3 ± 6.0 ab |
| *C. fioriniae* | 58.3 ± 2.9 e | 58.7 ± 2.3 f | 19.2 ± 7.3 a | 23.3 ± 2.1 a |
| *C. fructicola* | 66.3 ± 3.6 d | 71.1 ± 3.3 cd | 9.3 ± 2.1 bc | 20.6 ± 1.1 ab |
| *C. godetiae* | 63.0 ± 1.0 d | 48.7 ± 2.1 h | 9.0 ± 2.5 bc | 8.7 ± 3.9 de |
| *C. juglandicola* | 49.1 ± 1.9 f | 53.5 ± 3.0 g | 11.9 ± 3.0 abc | 14.7 ± 2.2 bcd |
| *C. karsti* | 70.5 ± 1.0 c | 69.0 ± 1.0 d | 6.8 ± 2.2 cd | 14.2 ± 5.6 bcd |
| *C. mengyinense* | 82.3 ± 1.5 a | 80.3 ± 0.6 a | 8.0 ± 2.1 bc | 19.4 ± 3.6 abc |
| *C. pandanicola* | 76.3 ± 4.3 b | 76.3 ± 1.5 ab | 17.1 ± 7.4 a | 22.4 ± 4.4 ab |
| *C. peakense* | 65.5 ± 0.9 d | 76.3 ± 2.6 ab | 6.8 ± 1.5 g | 10.4± 3.3 cd |
| *C. siamense* | 82.0 ± 0 a | 72.0 ± 3.5 bcd | 13.8 ± 6.6 ab | 21.8 ± 8.2 ab |
| CK | - | - | 0 ± 0 d | 0 ± 0 e |

^Z^ Data followed by different letters in each column are significantly different based on HSD tests at the *P*< 0.05 level.
